# Exosomes secreted by *ST3GAL5* ^high^ cancer cells promote peritoneal dissemination by establishing a premetastatic microenvironment

**DOI:** 10.1002/1878-0261.13524

**Published:** 2023-09-27

**Authors:** Misato Horie, Kurara Takagane, Go Itoh, Sei Kuriyama, Kazuyoshi Yanagihara, Masakazu Yashiro, Michinobu Umakoshi, Akiteru Goto, Junichi Arita, Masamitsu Tanaka

**Affiliations:** ^1^ Department of Molecular Medicine and Biochemistry Akita University Graduate School of Medicine Japan; ^2^ Department of Gastroenterological Surgery Akita University Graduate School of Medicine Japan; ^3^ Division of Rare Cancer Research National Cancer Center Research Institute Tokyo Japan; ^4^ Department of Molecular Oncology and Therapeutics Osaka Metropolitan University Graduate School of Medicine Japan; ^5^ Department of Cellular and Organ Pathology Akita University Graduate School of Medicine Japan

**Keywords:** exosomes, mesothelial cell, milky spots, peritoneal dissemination, ST3G5

## Abstract

Peritoneal dissemination of cancer affects patient survival. The behavior of peritoneal mesothelial cells (PMCs) and immune cells influences the establishment of a microenvironment that promotes cancer cell metastasis in the peritoneum. Here, we investigated the roles of lactosylceramide alpha‐2,3‐sialyltransferase (ST3G5; also known as ST3GAL5 and GM3 synthase) in the exosome‐mediated premetastatic niche in peritoneal milky spots (MSs). Exosomes secreted from *ST3G5* ^high^ cancer cells (ST3G5^high^‐cExos) were found to contain high levels of hypoxia‐inducible factor 1‐alpha (HIF1α) and accumulated in MSs via uptake in macrophages (MΦs) owing to increased expression of sialic acid‐binding Ig‐like lectin 1 (*CD169*; also known as *SIGLEC1*). ST3G5^high^‐cExos induced pro‐inflammatory cytokines and glucose metabolic changes in MΦs, and the interaction of these MΦs with PMCs promoted mesothelial–mesenchymal transition (MMT) in PMCs, thereby generating αSMA^+^ myofibroblasts. ST3G5^high^‐cExos also increased the expression of immune checkpoint molecules and T‐cell exhaustion in MSs, which accelerated metastasis to the omentum. These events were prevented following *ST3G5* depletion in cancer cells. Mechanistically, ST3G5^high^‐cExos upregulated chemokines, including CC‐chemokine ligand 5 (CCL5), in recipient MΦs and dendritic cells (DCs), which induced MMT and immunosuppression via activation of signal transducer and activator of transcription 3 (STAT3). Maraviroc, a C‐C chemokine receptor type 5 (CCR5) antagonist, prevented ST3G5^high^‐cExo‐mediated MMT, T‐cell suppression, and metastasis in MSs. Our results suggest ST3G5 as a suitable therapeutic target for preventing cExo‐mediated peritoneal dissemination.

## Graphical abstract

Exosomes secreted from *ST3G5* ^high^ cancer cells contribute to two major events in the premetastatic niche in peritoneal milky spots: transformation of peritoneal mesothelial cells into CAFs and immunosuppression. These events largely depended on the secretion of CCL5 from the exosome‐incorporated macrophages and dendritic cells. The CCL5–STAT3 pathway upregulated transcription factors necessary for mesothelial–mesenchymal transition, and induced immune checkpoint molecules and exhaustion in T cells.

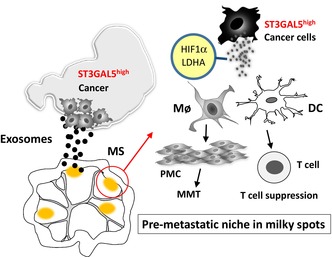

**Abbreviations**
7AAD: 7‐amino‐actinomycin D
CAF: cancer‐associated fibroblast
cExo: cancer cell‐derived exosomes
CM: conditioned medium
CRISPR: clustered regularly interspaced short palindromic repeats
EMT: epithelial‐mesenchymal transition
FACS: fluorescence‐activated cell sorting
H&E: hematoxylin and eosin
HIF1α: hypoxia‐inducible factor 1 subunit alpha
i.p: intraperitoneally
iDC: immature dendritic cells
LDHA: lactate dehydrogenase A
MFI: mean fluorescence intensity
MMT: mesothelial–mesenchymal transition
MS: milky spots
MΦ: macrophage
PDHE1α: pyruvate dehydrogenase‐E1 alpha
PMC: peritoneal mesothelial cell
s.c: subcutaneously
αSMA: alpha‐smooth muscle actin

## 1 Introduction

Advanced gastric cancer causes peritoneal dissemination, multiple metastasis to the peritoneum that affects survival. The peritoneum comprises peritoneal mesothelial cells (PMCs) supported by a thin layer of connective tissue [1], lines the abdominal cavity, and covers most abdominal organs. During peritoneal dissemination, cancer cells penetrate primary organs and expose to the outer surface, where they release exosomes into the abdominal cavity before detachment and metastasis to the distant peritoneum [2, 3]. Since these exosomes modify the behavior of PMCs and the immune microenvironment of the peritoneum, identifying the mechanism of exosome‐mediated establishment of the premetastatic microenvironment is essential.

Exosomes facilitate the communication between cancer and stromal cells in the tumor microenvironment. For example, the uptake of cancer cell‐derived exosomes (cExo) by stromal cells such as fibroblasts promotes cancer‐associated fibroblast (CAF)‐like differentiation [4]. Similarly, PMCs undergo a mesothelial–mesenchymal transition (MMT) via cExos and lose the barrier function, and participate in fibrosis [5]. We have previously reported that MMT of PMC was enhanced by macrophages (MΦs)‐incorporated cExo. Macrophages‐incorporated cExo of scirrhous gastric cancer cell line (44As3) induces prominent MMT compared with the direct effect of the same cExo on PMCs [6]. To identify the molecules responsible for this phenomenon, we observed that the mRNAs of genes related to synthesizing glycosphingolipids (GSL), such as gangliosides, are highly expressed in 44As3 cells.

Gangliosides, GSLs containing sialic acid residues and carried by exosomes [7, 8], are primarily located in lipid rafts of cell membrane, platforms for regulating transmembrane signal transduction [9]. Changes in ganglioside levels alter lipid rafts' molecular compositions and structures, thereby influencing cell functions [10]. Gangliosides are classified into four series based on the number of sialic acid residues, namely o‐, a‐, b‐, and c (Fig. S1A) [11]. Ceramide, indispensable for generating exosomes, is glucosylated to glucosylceramide and converted to lactosylceramide. GM3 is the first molecule in ganglioside family biosynthesis formed by the transfer of sialic acid to lactosylceramide by ST3G5 and regulates cell adhesion, proliferation, and migration [12]. For example, GM3 largely suppresses cell proliferation by blocking the activation of epidermal growth factor receptor and phosphoinositide‐3‐kinase–protein kinase B/Akt signaling [13].

ST3G5 expression has been reported to be elevated in some cancers [14, 15]. Its product, GM3, is a CD169/Siglec‐1 binding ligand, and GM3‐containing liposomes are incorporated by CD169^+^ MΦs [16], suggesting that ST3G5 in cancer cells affects the affinity of cExo to lymphoid tissues.

The omentum is the preferred early site of peritoneal metastasis of cancer cells, including gastric, pancreatic, and high‐grade serous ovarian cancer [17, 18, 19]. Milky spots (MS), lymphoid tissues most frequently observed in the omentum and mesentery, comprise B, T, and natural killer cells, along with MΦs, and other immune‐progenitor cells and play an essential role in cancer metastasis colonization [20, 21]. Previously, we performed cell lineage tracing of PMCs using a transgenic mouse (WT1^CreERT2^‐tdT), in which PMCs and their descendant cells were visualized using tdTomato fluorescent protein [22]. We applied this method to analyze the interaction between cExo‐incorporated MΦs and PMCs in MS. In this study, we observed that cExo of ST3G5^high^ cancer cells were preferentially incorporated into the MΦs of MS, further promoting CAF‐like transformation of PMCs.

The present study demonstrated that exosomes derived from ST3G5^high^ cancer cells activate MMT of PMCs via exosome‐incorporated MΦs and induced immunosuppression by inducing the immunocheckpoint molecules and T‐cell exhaustion. GM3 in ST3G5^high^‐cExo contributed to the uptake by MΦs and DCs, while hypoxia‐inducible factor 1‐alpha (HIF1α) and glycolytic enzymes, highly contained in ST3G5^high^‐cExo, upregulated inflammatory chemokines and lactate in recipient MΦs and dendritic cells (DCs), thereby suppressing T cells. In mouse models of peritoneal dissemination, ST3G5^high^‐cExos suppressed MS's immunoreactions, leading to exaggerated peritoneal metastasis of cancer cells. Our results suggest ST3G5 as a promising target for preventing peritoneal dissemination in some cancers.

## 2 Materials and methods

### 2.1 Cell lines and PMC preparation

The following cells were established from patients with gastric carcinoma as described [23, 24]: 44As3 (scirrhous‐type, RRID:CVCL_XG62), HSC‐44PE (scirrhous‐type, RRID:CVCL_A388), HSC‐43 (scirrhous‐type, RRID:CVCL_A387), HSC‐57 (intestinal‐type, RRID:CVCL_A613), HSC‐58 (scirrhous‐type, RRID:CVCL_A391), HSC‐59 (signet ring cell carcinoma, RRID:CVCL_A614), and HSC‐64 (poorly differentiated adenocarcinoma, RRID:CVCL_A617). MKN74 (intestinal‐type, RRID:CVCL_2791) and KATO‐III (signet ring cell carcinoma, RRID:CVCL_C1ND) were obtained from ATCC. HSC‐44PE and KATO‐III were established from pleural effusion. HSC‐57, HSC‐58, HSC‐59, and HSC‐64 were established from ascitic tumor. 44As3 was established from HSC‐44PE by repeated cycles of isolation of the ascitic tumor cells and orthotopic inoculation of these cells in BALB/c nude mice [23]. The following cells were obtained from ATCC: B16 (melanoma, RRID:CVCL_F936), PANC‐1 (RRID:CVCL_0480), Capan‐1 (RRID:CVCL_0237), CFPAC1 (RRID:CVCL_1119), BxPC‐3 (RRID:CVCL_0186), SUIT‐4 (RRID:CVCL_9280), A‐549 (RRID:CVCL_0023), H23 (RRID:CVCL_1547), H441 (RRID:CVCL_1561), H522 (RRID:CVCL_1567), H1703 (RRID:CVCL_1490), H1975 (RRID:CVCL_1511), H2126 (RRID:CVCL_1532), H2347 (RRID:CVCL_1550), LC‐2/ad (RRID:CVCL_1373), U‐87MG (RRID:CVCL_0022), 143B (RRID:CVCL_2270), SaOS‐2 (RRID:CVCL_0548), U2OS (RRID:CVCL_0042), HuO9 (RRID:CVCL_1298), MDA‐MB‐231 (RRID:CVCL_0062), and MCF7 (RRID:CVCL_0031). These authenticated cell lines were received within the last 3 years with a certificate. All cells were repeatedly screened for mycoplasma and maintained in culture for < 6 months after receipt. The identities of the cell lines were confirmed by STR analysis. Peritoneal mesothelial cells were isolated from rat mesenteries [25], and human CAFs (CAF‐37) were obtained from the tumoral gastric wall of scirrhous gastric cancer patients [25]. Peritoneal mesothelial cells and CAFs were cultured in DMEM containing 4500 mg·mL^−1^ glucose, 1 mm sodium pyruvate, 100 IU·mL^−1^ penicillin, 100 μg·mL^−1^ streptomycin, and 10% FBS. This study complies with the Declaration of Helsinki and is approved by the Osaka Metropolitan University Ethics Committee (approval number 2756, Osaka, Japan) and the Akita University Ethics Committee (approval number a‐1‐3175, Akita, Japan). All patients provided informed consent prior to the study. In some experiments, cells were cultured under hypoxia (O_2_, 1.0%) using a CO_2_‐multigas incubator (APM‐30D, ASTEC, Fukuoka, Japan).

### 2.2 Mice

C57BL/6 and BALB/c^nu/nu^ mice were obtained from CLEA. Wt1^CreERT2^‐tdTomato transgenic mouse was established by crossing Wt1‐Cre (Wt1^tm2(CreERT2)Wtp^/J) and ROSA26 (Jackson Laboratory, Bar Harbor, ME, USA) as described [22]. Wt1^CreERT2^‐tdTomato^nu/nu^ mice were prepared by crossing Wt1^CreERT2^‐tdT with BALB/c^nu/nu^ mice [22]. The mice were bred under specific pathogen‐free (SPF) conditions at the Animal Research Laboratory Bioscience Education‐Research Center of Akita University. All animal protocols were approved by the Committee for Ethics of Animal Experimentation (approval number a‐1‐3175, Akita, Japan), and the experiments were conducted in accordance with the Guidelines for Animal Experiments.

### 2.3 Exosomes

Cells were incubated in medium without FBS under the hypoxic condition for 48 h. In some experiments, 10% FBS previously depleted exosomes by ultracentrifugation at 100 000 ***g*** for 2 h, was added to the medium. The medium was collected, and exosomes were purified from the supernatants by ultracentrifugation as described previously [26]. Briefly, culture supernatants were cleared of cell debris by centrifugation at 300 ***g***, and centrifuged at 2000 ***g*** for 20 min to pellet large vesicles and apoptotic vesicles. The supernatant was centrifuged at 10 000 ***g*** for 30 min to remove microvesicles and then centrifuged again at 100 000 ***g*** for 2 h to pellet exosomes. In some experiments, isolated exosomes were labeled by ExoSparkler Exosome Membrane Labeling Kit (Green, Red) according to the manufacturers' instructions (Dojindo, Kumamoto, Japan).

### 2.4 Separation of biotin^+^ PMC by microbeads

Positive selection of biotin^+^ PMCs was performed using microbeads. Cells were magnetically labeled with streptavidin microbeads (Miltenyi Biotec, Bergisch Gladbach, Germany, 130‐048‐102), and passed through MACS MS columns (Miltenyi Biotec, 130‐042‐201), which were placed in the magnetic field according to the manufacturer's instruction. Biotin^+^ PMCs were flushed out from the column and passed over a new column to increase the purity. To remove contaminated MΦs, the samples were further passed through CD11b microbeads column (Miltenyi Biotec, 130‐049‐601) and anti‐F4/80 microbeads column (Miltenyi Biotec, 130‐110‐443).

### 2.5 Bone marrow‐derived DC induction

The bone marrow cells were freshly isolated from the tibia and femur of 6‐week‐old female C57BL/6 mice and maintained in RPMI‐1640 Medium (Sigma, St. Louis, MO, USA) supplemented with 10% heat‐inactivated FBS (PAA Laboratories, Pasching, Germany), 100 μg·mL^−1^ streptomycin, and 100 U·mL^−1^ penicillin (Sigma). To generate immature DCs (iDC), bone marrow cells were cultivated in the first 6 days with mouse GM‐CSF (Peprotech, Rocky Hill, UK, 315‐03) and IL‐4 (Peprotech, 214‐14; 25 ng·mL^−1^, each).

### 2.6 Flow cytometric analysis and cell sorting

CD3^+^ T cells were purified from 6‐week‐old female mouse spleens using MojoSort isolation kit (BioLegend, San Diego, CA, USA, CA 480024). In some experiments, isolated T cells were incubated with bone marrow‐derived DCs (in a ratio of 10 : 1) and the total cells were isolated and subjected to flow cytometric analysis with the mouse antibodies. The following antibodies are obtained by Miltenyi Biotec: Tim3 (RRID:AB_2654180), TIGIT (RRID:AB_2751782), PD‐1 (RRID:AB_2661365), CD152 (CTLA‐4; RRID:AB_2655253), CD69 (RRID:AB_2659092), CD25 (RRID:AB_2784091), FoxP3 (RRID:AB_2651767), CD86 (RRID:AB_2660747), IFN‐γ (RRID:AB_2819467), CD4 (RRID:AB_2752205), CD8 (PE; RRID:AB_2659884), CD11b (RRID:AB_2751174), CD3 (RRID:AB_2801803), and 7‐amino‐actinomycin D (7‐AAD; 130‐111‐568). The following antibodies are obtained by BioLegend: LAG‐3 (RRID:AB_2561516), PD‐L1 (RRID:AB_2073557), IL‐2 (RRID:AB_2561749), and MHC II (I‐A/I‐E; RRID:AB_893586). The following antibodies are obtained by TCI, Japan: GM3 (A2582) and GM2 (A2576). Anti‐GD3 (PRID:AB_669128) and anti‐GD2 (PRID:AB_1123587) were purchased from Santa Cruz Biotechnology, Dallas, TX, USA.

Cells were subjected to FACS analysis using a FACSMelody (BD Biosciences, San Jose, CA, USA) with FlowJo software (BD Biosciences, RRID:SCR_008520). In some experiments, exosomes were analyzed using PS capture Exosome Flow Cytometry Kit (FujiFilm, Tokyo, Japan, 297‐79701). To isolate tdTomato^+^ PMCs, dissociated cells were sorted by FACS (FACSMelody). Cell contamination was eliminated using FSC‐H and FSC‐W, SSC‐H, and SSC‐W. For positive and negative populations, the top 25% of stained cells or the bottom 20% of unstained cells were selected to be sorted, respectively. After collecting, the purity of the cell fraction was confirmed by fluorescence microscope.

### 2.7 Plasmids, antibodies, and reagents

The cDNA constructs encoding ST3G5 were amplified by RT‐PCR from the mRNA of 44As3 cells and sequenced. For expression in mammalian cells, cDNAs were cloned into pCS2+ (Addgene, Watertown, MA, USA) and the pCSII‐CMV‐MCS vector (RIKEN, Wako city, Japan). Synthetic DNA fragment of CD63 or MyrPalm (myristoylated and palmitoylated) was cloned into pEGFP‐N3 (Clontech, Santa Clara, CA, USA). Expression vector was transfected using Lipofectamine 2000 according to the manufacturer's instructions (Life Technologies, Carlsbad, CA, USA, 11668019). Recombinant lentiviral plasmids were co‐transfected along with packaging vectors into 293T cells to allow the production of the viral particles. HSC‐44PE cells stably expressing ST3G5 cDNA were established after viral infection by selection in medium containing puromycin.

Purchased antibodies were as follows: α‐tubulin (Sigma‐Aldrich, St. Louis, MO, USA, RRID:AB_477593), HIF1α (GeneTex, Irvine, CA, USA, RRID:AB_2616089), E‐cadherin (BD Biosciences, RRID:AB_397580), αSMA (Dako, Carpinteria, CA, USA, M0851), Mesothelin (Tecan (IBL), Mannedorf, Germany, RRID:AB_2148076), CD169 (Merck Millipore, Burlington, MA, USA, MABT328), p‐PDHE1α (Novus, Centennial, CO, USA, RRID:AB_1291446), GFP (ChromoTek, Planegg‐Martinsried, Germany, RRID:AB_2749857), CD8 (Bioss, Woburn, MA, USA, RRID:AB_10857537)，and F4/80 (Bio‐Rad, Hercules, CA, USA, RRID:AB_2098196). The following antibodies are obtained by Proteintech, Rosemont, IL, USA: ST3G5 (RRID:AB_2194414), Arg1 (RRID:AB_2289842), and PD‐L1 (RRID:AB_2756526). The following antibodies are obtained by Cell Signaling, Danvers, MA, USA: Akt (RRID:AB_329827), Phospho‐Akt (Ser473, RRID:AB_2315049), Erk1/2 (RRID:AB_330744), Phospho‐Erk1/2 (Thr202/Tyr204, RRID:AB_331646), Slug (RRID:AB_2239535), N‐cadherin (RRID:AB_10694647), Cleaved caspase‐3 (RRID:AB_2070042), pSmad2 (RRID:AB_490941), IL‐1β (RRID:AB_2715503), and CD25 (36128). The following antibodies are obtained by Santa Cruz Biotechnology: pSTAT3 (AB_628292), STAT3 (RRID:AB_632440), TLR4 (RRID:AB_10611320), CCL5 (RRID:AB_10844015), IL‐6 (RRID:AB_2127596), β‐actin (RRID:AB_2714189), LAG3 (sc‐514 993), LDHA (RRID:AB_2137192), and CD63 (RRID:AB_648179). For blocking experiments, anti‐mouse PD‐1 antibody was purchased from AdipoGen (AdipoGen Life Sciences, San Diego, CA, USA, AG‐20B‐0075PF) and the isotype control rat IgG2a was obtained from MBL (2H3, M081‐3).

ExoSparkler exosome membrane labeling kit (Dojindo, Red: 340‐09671, Green: 343‐09661), EZ‐Link Sulfo‐NHS‐LC‐biotin (membrane‐impermeable, Thermo Fisher Scientific, Waltham, MA, USA, 21335), GM3, and NGcGM3 (Cayman, Ann Arbor, MI, USA, GM3: 15587, NGcGM3: 33263), Alexa546‐streptavidin (Life Science, Waltham, MA, USA, S11225), Maraviroc (TargetMol, Boston, MA, USA, T6016).

### 2.8 Cytokine array analysis

To analyze the cytokines and chemokines in the CM of MΦs, the Proteome Profiler assay (ARY006, R&D Systems, Abingdon, UK) was used according to the manufacturer's instructions. Array images were acquired using ImageQuant LAS 500 (GE Healthcare, Chicago, IL, USA), and array signals from scanned images were quantified using imagej software (NIH, Bethesda, MD, USA).

### 2.9 Lactate assay

Lactate in the conditioned medium (CM) was quantified using Lactate Assay kit‐WST according to the manufacturer's instructions (Dojindo, L256). Lactate was quantified by measuring the absorption derived from a colorimetric reaction of water‐soluble Tetrazolium salts (WST) by a microplate reader (Multiskan FC, Thermo Fisher Scientific).

### 2.10 Cell cycle assay

Cell cycle assay was performed by quantitation of DNA content. Briefly, cells were fixed in cold 70% ethanol at 4 °C for 2 h, rinsed by PBS twice, and treated by RNase (0.25 μg·mL^−1^) at 37 °C for 30 min. Cells were then labeled by Propidium Iodide (BD Biosciences) and subjected to FACS analysis using a BD FACSAriaTM III (BD Biosciences) with facsdiva and bd flowjo software (BD Biosciences).

### 2.11 Tumor and tissue digestion

For the preparation of single‐cell suspensions from mouse tumors and tissues, specimens were mechanically dissociated and subsequently digested using, collagenase IV (Sigma‐Aldrich, 9001‐12‐1), Trypsin (Sigma‐Aldrich, T‐3924), and DNase type IV (Sigma‐Aldrich, 9003‐98‐9) for 30 min at 37 °C under constant agitation. Cell suspensions were filtered through 70‐μm mesh and lysed for red blood cells using BD Pharmlyse™ lysing buffer (BD Biosciences, 555899).

### 2.12 Gene targeting of ST3G5 by CRISPR/PITCH genome editing

Human and mouse ST3GAL5 genome sequences were edited using the PITCh method (Precise Integration into Target Chromosome) [27, 28]. The dataset of each genome editing was predicted by PITCh designer v2.0 (https://www.mls.sci.hiroshima-u.ac.jp/smg/PITChdesigner/index.html). The oligonucleotides of the target sequences of Cas9 were synthesized and inserted into GeneArt® CRISPR Nuclease Vector Orange Fluorescent Protein reporter vector (Thermo Fisher Scientific). The oligonucleotide sequences are listed in Table 1. The procedure for constructing the working vectors followed the company's manual. The method is based on the microhomology‐mediated end joining (MHEJ); thus, the neighbor sequences of PAM sequence (shown as left/right homology arm, Table 1) were used for the recombination of the target genome sequences with the target vectors. The cassette of CMV‐GFP‐P2A‐Neomycin‐resistant gene in the donor vector was inserted into the target genome region. The edited clones were briefly screened by the genomic PCR (primers are described in Table 1). And the clones of the potential candidates were analyzed by western blot.

**Table 1:** Sequences of sgRNA, left/right homology arm, and genomic PCR primers used for gene editing of mouse and human ST3G5. h, human; m, mouse.

mST3G5_sgRNA_Fw/Rv	5′‐TTTGCAGAGGTGAACTCACTGTTTT‐3′	5′‐AGTGAGTTCACCTCTGCAAACGGTG‐3′
Left/Right homology arm_mST3GAL5	GTGATTGCTCAAGGACCTCCNNNNNNCAATGGTACACCCGAACCCA	
mST3G5 genomic DNA primers	5′ACCTAGCTGCTGTTGATCTAGAGGGGTC‐3′	5′‐ATCGCATTGTCTGAGTAGGTGTCATTCTATTC‐3′
hST3G5_sgRNA_Fw/Rv	5′GACGAAGGCGGCGGGCTGCGGTTTT‐3′	5′‐CGCAGCCCGCCGCCTTCGTCCGGTG‐3′
Left/Right homology arm_hST3GAL5	TGCGGACGAAGGCGGCGGGCNNNNNNGCGGAGCGGCGTCCCCTGCA	
hST3G5 genomic DNA primers	5′‐TCATTAGTATGCGGACGAAGGCGGC‐3′	5′‐CCGCTGGTAGAAATTTGCCGGTGA‐3′
PITCh target_sgRNA	5′‐GCATCGTACGCGTACGTGTT‐3′	

### 2.13 Gene‐expression profiling

Total RNAs were extracted from cultured mouse iDCs with or without treatment by cExo using Pure Link RNA mini Kit (Invitrogen, Waltham, MA, USA). Separately, total RNAs were extracted from tissues of MSs collected from mice with or without injection of cExo. These RNAs were subjected to RNA sequence analysis (Macrogen, Sakuragaoka, Tokyo, Japan). Data were analyzed using the Microarray Data Analysis Tool Ver3.2 (Filgen, Nagoya city, Japan) and DAVID Bioinformatics Resources 6.8 (Laboratory of Human Retrovirology and Immunoinformatics).

### 2.14 Quantitative real‐time PCR analysis and RT‐PCR

Total RNA was extracted from the indicated cells using the Pure Link RNA mini Kit (Invitrogen), and cDNA was produced from 1 μg RNA by reverse transcription, according to the manufacturer's recommendations (Roche Diagnostics, Kounan, Tokyo, Japan). Quantitative PCR was conducted in a LightCycler Nano using a SYBR Green kit (Roche Diagnostics, Kounan, Tokyo, Japan). Samples were normalized against GAPDH. A list of primers are as follows:

mCCL5‐Fw: 5′‐CCTCACCATATGGCTCGGAC‐3′,
mCCL5‐Rv: 5′‐TCTTCTCTGGGTTGGCACAC‐3′,
mCCL22‐Fw: 5′‐CCTGATGACCATGGGTCTGG‐3′,
mCCL22‐Rv: 5′‐TGGGGAGTAGGCATTGGGTA‐3′,
mIL‐1β‐Fw: 5′‐TGCCACCTTTTGACAGTGATG‐3′,
mIL‐1β‐Rv: 5′‐AAGGTCCACGGGAAAGACAC‐3′,
mIL‐6‐Fw: 5′‐ACTTCACAAGTCGGAGGCTT‐3′,
mIL‐6‐Rv: 5′‐TGGTCCTTAGCCACTCCTTCT‐3′,
mGAPDH‐Fw: 5′‐TTTGGCTACAGCAACAGGGT‐3′,
mGAPDH‐Rv: 5′‐ACCCTGTTGCTGTAGCCGTAT‐3′,
rACTA2 (αSMA)‐Fw: 5′‐CATCCGACCTTGCTAACGGA‐3′,
rACTA2 (αSMA)‐Rv: 5′‐AATAGCCACGCTCAGTCAGG‐3′,
rGAPDH‐Fw: 5′‐AGTGCCAGCCTCGTCTCATA‐3′,
rGAPDH‐Rv: 5′‐ACCAGCTTCCCATTCTCAGC‐3′.

### 2.15 Immunoblotting

Cell lysates were prepared in PLC buffer [50 mm HEPES (pH 7.5), 150 mm NaCl, 1.5 mm MgCl_2_, 1 mm EGTA, 10% glycerol, 100 mm NaF, 1 mm Na_3_VO_4,_ and 1% Triton X‐100] containing protease inhibitors. Cell lysates were separated by SDS/PAGE and immunoblotted. All primary antibodies were used at dilutions of 1 : 1000.

### 2.16 *In vivo* tumor transplantation

Specific pathogen‐free BALB/c^nu/nu^ mice and C57BL/6 mice (6‐week‐old, males) were obtained from CLEA Japan, Inc. (Higashiyama, Tokyo, Japan). The mice were bred under SPF conditions at the Animal Research Laboratory Bioscience Education‐Research Center of Akita University. All animal experimental protocols were approved by the Committee for Ethics of Animal Experimentation (approval number a‐1‐3175, Akita, Japan), and the experiments were conducted in accordance with the guidelines for Animal Experiments at Akita University. On the indicated days, mice were sacrificed by cervical dislocation. Five mice were used per group, and randomization was not performed. When assessing the outcome, the investigators were blinded to the group allocation.

### 2.17 Specimens from cancer patients

Gastric cancer specimens were obtained from 24 patients who had undergone resection of primary gastric tumors. None of the patients had undergone preoperative radiation or chemotherapy. All samples were collected in Akita University Hospital, Akita, Japan, between January 2009 and December 2020 and tissues were obtained with the written informed consent of the patients. Clinicopathologic findings from these patients are summarized in Table 2. The study was approved by the Akita University Ethics Committee (approval number 1662, Akita, Japan).

**Table 2:** Correlation between ST3G5 expression and clinicopathological features in gastric cancer.

	ST3GAL5 expression		Total numbers
	Negative (*n* = 17)	Positive (*n* = 7)	
Age			
< 60	5 (71%)	2 (29%)	7
> 60	12 (71%)	5 (29%)	17
Gender			
Male	9 (69%)	4 (31%)	13
Female	8 (73%)	3 (27%)	11
Tissue type			
Scirrhous type	8 (73%)	3 (27%)	11
Nonscirrhous type	9 (69%)	4 (31%)	13
Invasion depth			
T1, T2	10 (83%)	2 (17%)	12
T3, T4	7 (58%)	5 (42%)	12
Regional lymph nodes			
N0	10 (90%)	1 (10%)	11
N1	1 (33%)	2 (67%)	3
N2	3 (75%)	1 (25%)	4
N3	3 (50%)	3 (50%)	6
Lymphatic invasion			
ly0	3 (100%)	0 (0%)	3
ly1 ~ 3	14 (67%)	7 (33%)	21
Venous invasion			
v0	6 (100%)	0 (0%)	6
v1 ~ 3	11 (61%)	7 (39%)	18
Peritoneal recurrence			
Negative	10 (91%)	1 (9%)	11
Positive	7 (54%)	6 (46%)	13

### 2.18 Immunohistochemical analysis

Paraffin blocks were sectioned and subjected to immunohistochemical staining using the Envision reagent (Dako, K4002). Antigen retrieval was performed by placing sections in Target retrieval solution (Dako, PH6 S1699, PH9 S2367) and heating to 95 °C in a water bath, according to the manufacturer's instructions. For the immunostaining of human gastric cancer specimens, anti‐ST3G5 antibody (Proteintech) was used at dilutions of 1 : 400. In co‐immunostaining experiments, sections were sequentially stained with each antibody using an Opal™ four‐color IHC Kit and fluorescently conjugated tyramide according to the manufacturer's instructions (PerkinElmer, Waltham, MA, USA, NEL794001KT). Horseradish peroxidase (HRP)‐conjugated secondary antibody (GE Healthcare, anti‐mouse IgG: NA931, anti‐rabbit IgG: NA934) was added for 10 min, and incubated with Opal kit working solution including the desired fluorophore. Tissues underwent the microwave treatment for removal of primary and secondary antibodies before another round of staining according to the Opal Multiplex IHC Assay Development Guide and Image Acquisition Information (Akoya Biosciences, Menlo Park, CA, USA).

### 2.19 Immunofluorescence staining

Cells were fixed with 4% paraformaldehyde in PBS and permeabilized for 5 min with 0.1% Triton X‐100. Cells were preincubated in 5% bovine serum albumin for 30 min and incubated with specific primary antibodies for 1 h at room temperature. After washing, cells were incubated with Alexa Fluor‐conjugated secondary antibodies (Invitrogen) for 1 h at room temperature. Images were obtained using an LSM780 or LSM980 (Zeiss, Oberkochen, Germany) confocal microscope and processed using zen software (Zeiss).

### 2.20 Gene set analysis of database

RNA sequencing dataset GSE162214 in Gene Expression Omnibus (GEO) was analyzed. First, ST3GAL5 gene expression was examined in GSE162214 by GEO2R software (NCBI), and the highest five and lowest five samples were selected. The counts of gene‐expression profiles of those selected datasets were further analyzed by RNAseqChef web‐based transcriptome analysis.

### 2.21 Statistical analysis

Data are expressed as the mean ± standard deviation. Statistical significance was calculated using the Student's *t*‐test. *P*‐values < 0.05 were considered statistically significant.

## 3 Results

### 3.1 ST3G5 expression in cancer cells regulates hypoxia‐inducible factor 1‐alpha (HIF1α) and downstream target molecules.

44As3 is a scirrhous gastric cancer cell line with a high potential for peritoneal dissemination established from the parent HSC‐44PE cells with low dissemination [23]. We previously observed that MΦs‐incorporated exosomes of 44As3 cells significantly altered PMCs to mesenchymal phenotypes and promoted peritoneal dissemination [6]. In this study, we focused on ST3G5 (GM3 synthase), which was elevated in 44As3 cells compared with HSC‐44PE cells, to examine the exosome‐mediated increase in peritoneal dissemination (Fig. S1B).

**Fig. 1:**
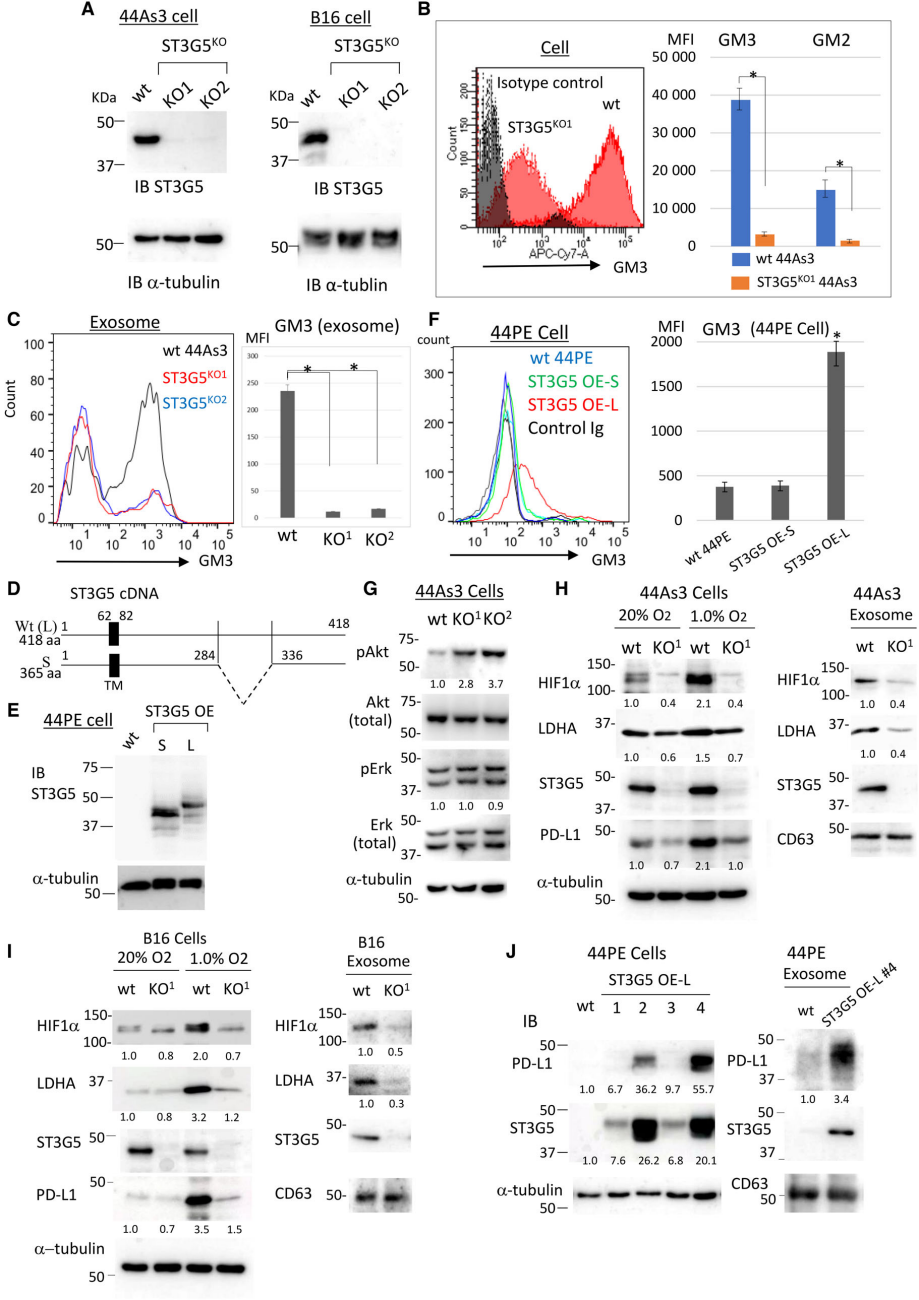
Hypoxia‐inducible factor 1‐alpha (HIF1α) and its target molecules are upregulated in ST3G5^high^ cancer cells. (A) ST3G5 gene was targeted in 44As3 and B16 cells, and repeatedly checked by immunoblot analysis (*n* = 4). KO1 and KO2 indicate the two independent clones after selection. (B) GM3 and GM2 in the wild‐type (wt) or ST3G5^KO1^ 44As3 cells were evaluated by flow‐cytometer, and the mean fluorescence intensity (MFI) was compared. (C) GM3 amount in exosomes was analyzed by FACS. (D) Schematic diagram of human ST3G5 cDNA. TM, transmembrane. (E) Representative images of immunoblotting (*n* = 2) of ST3G5 in HSC‐44PE cells expressing short (S) or wt (L) ST3G5 cDNA. (F) FACS analysis of GM3 in wild‐type or ST3G5 overexpressed (OE) HSC‐44PE cells. B, C, F; mean ± SD, five independent experiments. *P < 0.01, Student's *t*‐test. (G) Activation of Akt and Erk was examined in 44As3 cells by immunoblotting (*n* = 2, representative results). (H, I) Wild‐type or ST3G5^KO^ cells of 44As3 (H) or B16 (I) were incubated in the standard or hypoxic condition, and exosomes were collected from hypoxic condition. Lysates were prepared from the cells and exosomes and subjected to immunoblot analysis. Western blot of each cell lysate was performed from the three independent experiments, and the statistical analysis was shown in Fig. S2B. (J) Immunoblot analysis of PD‐L1 in HSC‐44PE cell clones overexpressing wt ST3G5 at various levels. (G–J) The intensities of each band were quantified, and normalized by α‐tubulin. Expression of phosphorylated Akt or Erk was further adjusted by each total protein. The relative ratios were described below the panel.

To elucidate the effects of ST3G5, gene targeting of ST3G5 was performed in 44As3 cells and B16 mouse melanoma cells to confirm some results in different cancers (Fig. 1A). The amount of GM3 was significantly reduced in 44As3 ST3G5^KO^cells (Fig. 1B). In addition, GM2, synthesized from GM3, was also downregulated (Fig. 1B, right), while GD3 and GD2 were not expressed in 44As3 cells (Fig. S1C). GM3 was detected in exosomes of 44As3 cells, which was significantly reduced in ST3G5^KO^ 44As3‐cExo (Fig. 1C).

When ST3G5 cDNA was amplified from 44As3 cell mRNA, wild‐type (L) and truncated isoforms, in which 156‐bp nucleotides were deleted within the glycol transferase domain near the C terminus (ST3G5‐S, corresponding to Uniprot: A0A1W2PR45), were identified (Fig. 1D,E). Transduction of ST3G5‐S in HSC‐44PE cells, which do not express endogenous ST3G5, revealed that GM3 levels were solely increased by ST3G5‐L cDNA (Fig. 1F). Tumors of ST3G5^KO^ 44As3 cells in nude mice were more extensive than those of wt 44As3 cells (Fig. S2A). Accordingly, activation (phosphorylation) of Akt was elevated in ST3G5^KO^ 44As3 cells, as expected (Fig. 1G).

Since 44As3 cells express high levels of HIF1αand molecules related to anaerobic glycolysis [29], they were examined in ST3G5^KO^ cells. The induction of HIF1αand lactate dehydrogenase A (LDHA), a regulator of glycolysis, was attenuated under hypoxia by ST3G5 depletion (Fig. 1H, Fig. S2B). HIF1α and LDHA were observed in wt 44As3‐cExo, which was attenuated in ST3G5^KO^ cells (Fig. 1H, right). The amount of HIF1αand LDHA was also reduced in ST3G5^KO‐^B16 cells under hypoxia (Fig. 1I, left, Fig. S2B) and their exosomes (Fig. 1I, right). The expression of programmed death‐ligand 1 (PD‐L1), which is upregulated by HIF1α [30], was elevated in 44As3 cells and B16 cells, particularly under the hypoxic condition, and reduced by ST3G5 depletion (Fig. 1H,I, left). Although PD‐L1 was not clearly detected in exosomes of these cells, it was increased in ST3G5 overexpressed (OE) HSC‐44PE cells based on the level of ST3G5 (Fig. 1J, left), and PD‐L1 protein was detected in the exosomes of ST3G5‐OE 44PE cells (Fig. 1J, right). These results suggest that ST3G5 in cancer cells modifies the contents in the exosomes, that is, HIF1α and LDHA, which may affect the HIF1α‐mediated signaling in the recipient cells.

### 3.2 MΦs‐incorporated ST3G5^high^‐cExo contribute to mesothelial cells for CAF transition

**Fig. 2:**
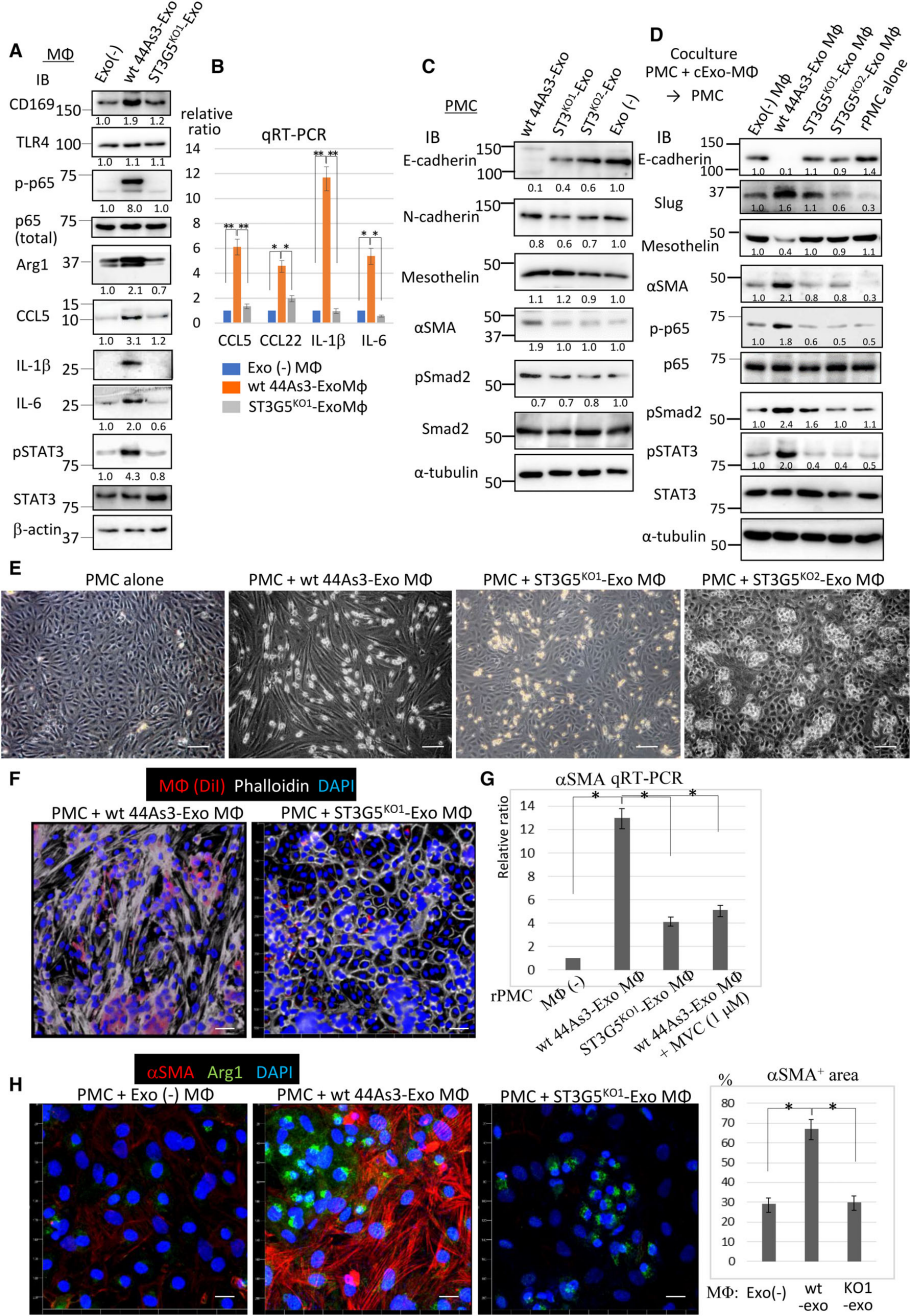
Incorporation of ST3G5^high^‐cExo into macrophages accelerated mesothelial–mesenchymal transition (MMT). (A, B) Macrophages (MΦs) were treated by exosomes of 44As3 cells (wt or ST3G5^KO^ clones; 5 μg·mL^−1^) for 2 days, and the cells were subjected to immunoblot analysis (A, representative results, *n* = 2), or quantitative real‐time PCR (qRT‐PCR; B, *n* = 3). (B) Mean ± SD, *P < 0.05, **P < 0.01, Student's *t*‐test. (C) Rat peritoneal mesothelial cells (PMCs) were treated by 44As3‐cExos as above for 5 days. Cell lysates were prepared for immunoblot analysis (*n* = 2). (D) Cell surface total proteins of PMCs were labeled by membrane‐impermeable biotin. MΦs were incubated with exosomes as above for 2 days, and mixed with the biotin‐labeled PMCs (1 : 1 Ratio) and co‐cultured for 3 days. Biotin^+^ cells were collected using streptavidin mag‐beads column, and then, the contaminated MΦs were sequentially removed by CD11b and F4/80 mag‐beads columns. Biotin^+^/CD11b^−^/F4/80^−^ cells represent PMCs and subjected to immunoblot analysis (*n* = 2). (A, C, D) The intensities of each band were quantified and normalized by β‐Actin or α‐tubulin. The relative ratios were described below the panel. (E) Representative optical microscope images of PMCs cultured alone or with MΦs‐incorporated wt or ST3G5^KO^ 44As3‐cExo (5 μg·mL^−1^). Bar, 50 μm. *n* = 3. (F) Co‐culture was performed as above after the cExo‐uptake MΦs were labeled by DiI (red; *n* = 3). The cells were fixed and immunostained with phalloidin and DAPI. Bar, 50 μm. (G, H) Expression of αSMA in PMC was analyzed by qRT‐PCR (G, *n* = 3) or immunofluorescence staining (H, Bar: 20 μm). In G, PMCs and MΦs were derived from rat and mouse, respectively; all primers used for PCR were designed from the rat sequence (i.e., they do not amplify the corresponding mouse gene). MVC: Maraviroc. In H, the percentage of the area of αSMA^+^ cells in the total cell area was calculated in three independent images and summarized (right panel). (G, H) Mean ± SD, *P < 0.01, Student's *t*‐test.

We next examined molecular changes in cExo‐uptake MΦs (cExo‐MΦs). In wt 44As3‐cExo‐MΦs, we observed an elevation in CD169, an adhesion molecule that binds to GM3 [31], and Arg1, a marker of M2 MΦ [32], while TLR4, which interacts with GM3, remained unchanged (Fig. 2A) [33]. Wt 44As3‐cExo activated NF‐_k_B (p65) in MΦ, thereby upregulating inflammatory cytokines and chemokines, including interleukin (IL)‐6, IL‐1β, and CC‐chemokine ligand 5 (CCL5; Fig. 2A,B). As a downstream molecule, activation (phosphorylation) of signal transducer and activator of transcription 3 (STAT3) was detected in the same cell lysates (Fig. 2A). These events were attenuated in MΦs receiving ST3G5^KO^ 44As3‐cExo (Fig. 2A). We also examined the CM of these MΦs by cytokine array analysis and revealed that CCL2, TIMP‐1, and IL‐12 were also elevated in the CM of MΦs treated by Wt 44As3‐cExo (Fig. S2C). On the contrary, secretion of IFN‐γ was rather decreased by Wt 44As3‐cExo (Fig. S2C).

In addition, wt 44As3‐cExo increased expression of molecules involved in anaerobic glycolysis including LDHA, which produces lactic acid, and phosphorylated pyruvate dehydrogenase‐E1α, restricting the synthesis of acetyl‐coenzyme A (Fig. S2D) [34]. Flow cytometric analysis revealed that wt 44As3‐cExo increased PD‐L1 expression in MΦs, which is induced by glycolytic metabolic reprogramming (Fig. S2E) [35]. These events were again attenuated in MΦs receiving ST3G5^KO^ 44As3‐cExo (Fig. S2D,E), suggesting that exosomes of ST3G5^high^ cancer cells reprogram MΦs for glycolytic metabolism, which upregulates PD‐L1. We also observed similar changes in MΦs receiving wt or ST3G5^KO^ B16‐cExo (Fig. S2F).

Peritoneal mesothelial cells serve as a protective anatomical barrier during the peritoneal dissemination of cancer cells. When cultured PMCs were directly treated with wt or ST3G5^KO^ 44As3‐cExos, a decrease in E‐cadherin was detected by wt 44As3‐cExo, whereas expression of mesothelin (MC marker) was not altered and αSMA (CAF marker) was slightly elevated (Fig. 2C). However, when PMCs were co‐cultured with MΦs‐incorporated wt 44As3‐cExo (wt 44As3‐cExo‐MΦ), MMT was characterized by the reduction in E‐cadherin and mesothelin, while the elevation of Slug and αSMA was more evident (Fig. 2D). Activation of NF‐κB and downstream STAT3 was also observed (Fig. 2D). Mesothelial–mesenchymal transition was undetected by ST3G5^KO^ 44As3‐cExo‐MΦs or untreated MΦs (Fig. 2D), yet was detected after co‐culture with MΦ‐incorporated ST3G5‐overexpressing HSC‐44PE‐cExo (Fig. S2G).

Morphologically, the epithelial sheet of PMC took on a spindle‐shaped, fibroblastic appearance following co‐culture with wt 44As3‐cExo‐MΦs, which indicated MMT (Fig. 2E,F). Consistently, αSMA was induced in PMCs by wt 44As3‐cExo‐MΦs (Fig. 2G,H). These events were not evident in the co‐culture with ST3G5^KO^ 44As3‐cExo‐MΦs (Fig. 2E–H).

Inflammatory signaling contributes to the epithelial‐mesenchymal transition (EMT) [36], which may explain MMT in co‐culture with wt 44As3‐cExo‐MΦs. Herein, we focused on CCL5, which is known to induce EMT in various epithelial cells [37]. Treatment with Maraviroc, which blocks the interaction of CCL5 and C‐C chemokine receptor type 5 (CCR5), of the co‐culture of PMCs and wt 44As3‐cExo‐MΦs attenuated MMT (Fig. 2G, Fig. S2H), suggesting that CCL5 secretion from these MΦs may be a major contributing cause of MMT.

### 3.3 ST3G5^high^‐cExo causes MMT in MS and accelerates peritoneal metastasis

**Fig. 3:**
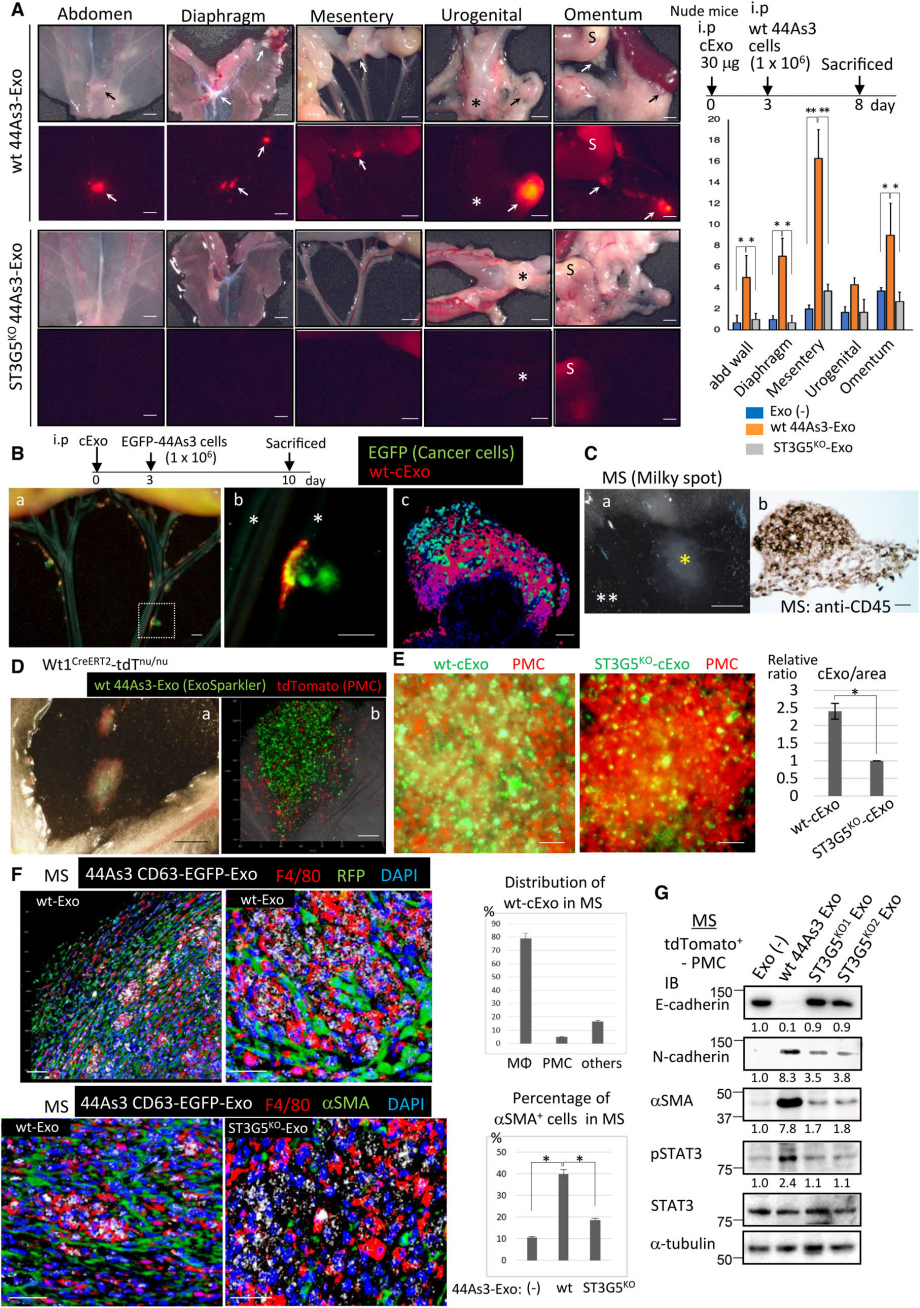
ST3G5^high^‐cExos accumulate in milky spots (MS) and cause mesothelial–mesenchymal transition (MMT). (A) Exosomes of wt or ST3G5^KO^‐44As3 cells were intraperitoneally (i.p) injected or not injected (Exo‐) in nude mice (30 μg per body), and DiI‐labeled wt 44As3 cells were i.p injected on Day 3. Mice were sacrificed on Day 8. Representative appearance were shown by dissecting microscope (upper panels) or fluorescence microscope (bottom panels). Tumor nodules were indicated by arrows. Asterisk: bladder, S: stomach. Bar, 2.5 mm. The number of the tumor was summarized in the right. Five mice were examined in each group and repeated for two times. Mean ± SD, *P < 0.01, **P < 0.006, Student's *t*‐test. (B) Wt 44As3‐cExo (30 μg) were labeled by ExoSparkler (red), and i.p injected in nude mice, followed by i.p injection of EGFP‐labeled 44As3 cells. Representative appearance of the mesentery (*n* = 4). Boxed area in a was enlarged in b. Bar, 1 mm. Weak green color in vessels (asterisk) is the auto‐fluorescence background. (c) Mesentery was fixed and embedded in the compound, and frozen sections were prepared. Representative tumor nodule was shown. Bar, 200 μm. (C) (a) Milky spots (yellow asterisk) in the mesentery sheet (white asterisks). Bar, 1 mm. (b) MS were fixed, paraffin‐embedded, and immunostained with anti‐CD45 antibody. Bar, 200 μm. Representative image of five MS. (D) ExoSparkler‐labeled Wt 44As3‐cExo (green, 30 μg) were i.p injected in WT1^CreERT2^‐tdT^nu/nu^ mice (*n* = 5). WT1 promoter regulated tdTomato, represents mesothelial cells (red). Representative appearance of the MS in mesentery on Day 3. Bar, 1 mm. (b) Bar, 200 μm. (E) Representative enlarged images of MS in mice injected Wt or ST3G5KO‐44As3 cExo, as described in (D). Bar, 50 μm. Right: summary of each five fields. Mean ± SD, *P < 0.01, Student's *t*‐test. (F) Exosomes of 44As3 cells expressing CD63‐EGFP (upper; Wt 44As3, bottom; Wt or ST3G5^KO^ 44As3) were i.p injected in WT1^CreERT2^‐tdT^nu/nu^ as above (*n* = 5 in each). Paraffin sections of MS were subjected to multi‐colored fluorescence immunostaining as indicated. CD63‐EGFP^+^ exosomes were visualized by anti‐EGFP antibody (white), and tdTomato was detected by anti‐RFP antibody (upper panels, green). Bar, 50 μm. Right: Summary of each five mice. Mean ± SD, *P < 0.01, Student's *t*‐test. (G) Wt or ST3G5^KO^ 44As3‐cExos were i.p injected as above, and MSs were collected. TdTomato expressing PMCs were sorted from dispersed cells of the MS by FACS and subjected to immunoblot analysis. Experiments were repeated three times, and representative results are shown.

We next examined the effects of ST3G5^high^‐cExo on modification of peritoneal microenvironment prior to metastasis. Wild‐type or ST3G5^KO^ 44As3‐cExos were labeled with ExoSparkler and injected (i.p) into nude mice before inoculation with 44As3 parent cells (Fig. 3A, right scheme). The number of tumor nodules in various organs in the peritoneal cavity was more significant in those preinjected wt 44As3‐cExo than in mice preinjected ST3G5^KO^ 44As3‐cExo or without exosomes (Fig. 3A, Fig. S3A).

These observations suggest that ST3G5^high^‐cExo prepared a premetastatic niche in the peritoneum. To visualize the location of cExo and cancer cells, ExoSparkler‐labeled 44As3‐cExos and EGFP‐labeled 44As3 cells were sequentially injected (i. p.) into nude mice, which were sacrificed 7 days later (Fig. 3B). EGFP‐44As3 cells accumulated in the spots of preinjected cExos (Fig. 3Ba–c), which most likely correspond to MS, mainly present on the mesentery, particularly along the blood vessels (Fig. 3Ba,b), and omentum as vague structures (Fig. 3Ca).

We have previously performed lineage tracing of PMCs using Wt1^CreERT2^‐tdTomato^nu/nu^ (designated Wt1^CreERT2^‐tdT^nu^) mice, in which PMCs were visualized using tdTomato [22]. When ExoSparkler‐labeled cExos were injected into these mice, they were localized in the spots where PMCs accumulated (Fig. 3D). These spots contained dense CD45^+^ immune cells, indicating MS (Fig. 3Cb). When the amounts of 44As3‐cExos in the MS were evaluated, more wt‐cExos were detected than ST3G5^KO^‐cExos (Fig. 3E). cExos were mainly incorporated into the MΦs of MS (Fig. 3F, upper).

We also observed α SMA‐positive fibroblastic cells surrounding cExo‐MΦs, which was less extent in the MS treated with ST3G5^KO^ 44As3‐cExo (Fig. 3F, bottom). The MMT of PMCs in MS was further examined by collecting the tdTomato^+^ PMCs via FACS. The gain of N‐cadherin, αSMA, and activation of STAT3 accompanied the decrease in E‐cadherin expression was detected in PMCs by inoculation with wt 44As3‐cExo, but not ST3G5^KO^ 44As3‐cExo (Fig. 3G). These results support that ST3G5^high^‐cExo‐uptake MΦs caused MMT in MS, and served the premetastatic niche.

### 3.4 Upregulation of CD169 by ST3G5^high^‐cExo accelerates exosome uptake in MΦ.

**Fig. 4:**
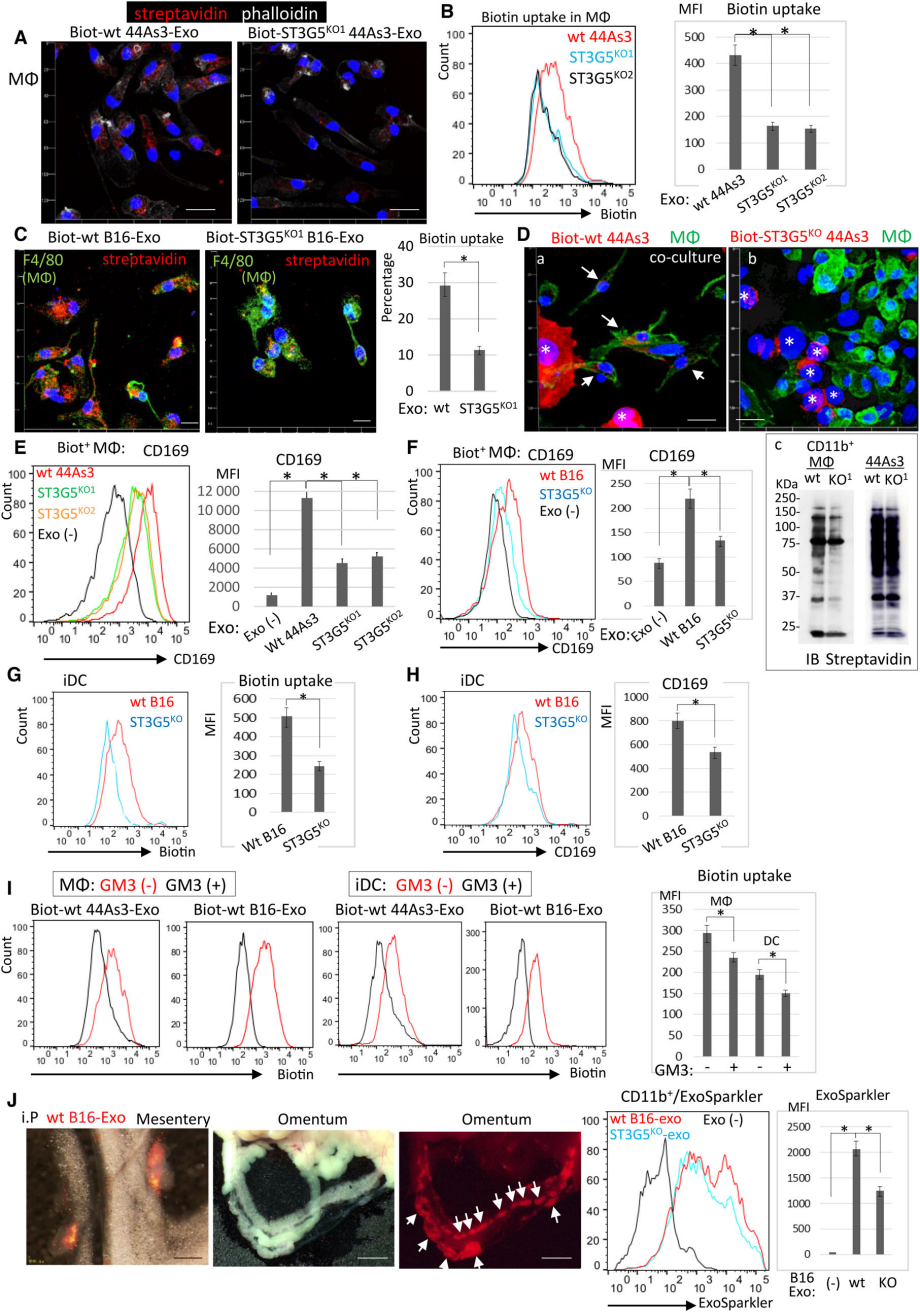
ST3G5^high^‐cExo augments CD169 expression in MΦs, which accelerates exosome uptake. (A–C) Cell surface proteins of wt or ST3G5^KO^ 44As3 cells (A, B) or B16 cells (C) were labeled by membrane‐impermeable biotin, and exosomes were collected, added to MΦs (cExo; 5 μg·mL^−1^), and incubated for 12 h. The MΦs were fixed and immunofluorescence stained by streptavidin and phalloidin (A, representative images of five fields in each), or subjected to FACS analysis of biotin (B). (C) Uptake of biotin‐labeled B16‐cExo in MΦs was assessed by immunostaining. Biotin incorporated in MΦs was expressed as the percentage of red fluorescence area in each cell. Random 100 cells were analyzed. (D) Biotin‐labeled wt (a) or ST3G5^KO^ (b) 44As3 cells were mixed with MΦs and co‐cultured for 24 h. Cells were fixed, and immunofluorescence stained by anti‐CD11b antibody (green) and streptavidin (red). Representative images of the co‐culture with 44As3 were shown. Arrows indicate biotin incorporated MΦs. Asterisks indicated biotin‐labeled 44As3 cells. Bar, 100 μm. (c) Co‐cultured cells were collected, and CD11b^+^ MΦs were sorted by FACS, and subjected to immunoblot analysis of biotin amount. Larger amount of biotin^+^ proteins of wt 44As3 cell was transferred into MΦs (left panel). Equal amount of biotin‐labeled proteins was observed in wt and ST3G5^KO^‐44As3 cells (right panel). (E, F) MΦs were incubated with biotin‐labeled 44As3‐cExo (E) or B16‐cExo (F) (5 μg·mL^−1^), and CD169 expression in biotin‐uptake MΦs was analyzed. (G, H) Biotin^+^ B16‐cExos were added to immature DCs prepared from bone marrow for 2 days (5 μg·mL^−1^), and biotin‐uptake and CD169 expression were analyzed as above. (I) MΦs or iDC were incubated with biotin^+^ exosomes of wt‐44As3 or ‐B16 cells (5 μg·mL^−1^) with/without soluble GM3 (20 μm). Incorporated biotin was evaluated by FACS. (J) C57BL/6 mice were i.p injected by wt or ST3G5^KO^ B16‐cExo labeled with ExoSparkler (red, 30 μg). As the control, exosomes were not injected (Exo(−)). Representative appearance of the mesentery and omentum. Arrows indicate the cExo in MS. Bar, 1 mm (mesentery), 2.5 mm (omentum). Right: MΦs were gated by CD11b^+^, and ExoSparkler was evaluated. Five mice were examined in each group. B, E–I; Results of three independent experiments. B, C, E–J; mean ± SD. *P < 0.01, Student's *t*‐test.

The efficiency of cExo uptake affects the function of MΦ. Biotin‐labeled wt 44As3‐cExo was more efficiently incorporated into MΦs than ST3G5^KO^ 44As3‐cExo (Fig. 4A,B). Similarly, cExo of ST3G5^KO^‐B16 cells was less incorporated to MΦs (Fig. 4C). Cell‐contact‐dependent transfer of cancer cell molecules to MΦ was also augmented by ST3G5 expression in cancer cells. When wt 44As3 cells, in which cell surface proteins, were labeled by biotin were mixed with MΦs, biotin^+^ proteins were incorporated into MΦs (Fig. 4D, left). Such transfer was greatly reduced by the co‐culture with ST3G5^KO^ 44As3 cells, which was evaluated as shown by the immunoblot analysis of biotin^+^ proteins in co‐cultured MΦs (Fig. 4D, right). These results suggest that cell surface GM3 may also increase cell‐contact‐dependent transfer of cancer cell molecules to MΦs.

We confirmed that CD169 expression was upregulated in MΦs‐incorporated wt 44As3‐ or B16‐cExo compared with ST3G5^KO^‐cExo (Fig. 4E,F). Similar results were obtained in iDC prepared from bone marrow cells (Fig. 4G,H). Moreover, adding recombinant GM3 at least partially attenuated the uptake of cExo, further suggesting a GM3/CD169‐dependent increase in exosome uptake by MΦ and iDC (Fig. 4I).

Next, the incorporation of exosomes was compared in mice MS. ExoSparkler‐labeled wt or ST3G5^KO^ B16‐cExo (25 μg, each) was intraperitoneally injected into the syngeneic mice, and MS in the mesentery and omentum were collected 3 days later (Fig. 4J). FACS analysis revealed that a more significant amount of wt B16‐cExo was detected in MS MΦs (Fig. 4J, right).

### 3.5 ST3G5^high^‐cExo induces immune checkpoint molecules and exhaustion in T cells

**Fig. 5:**
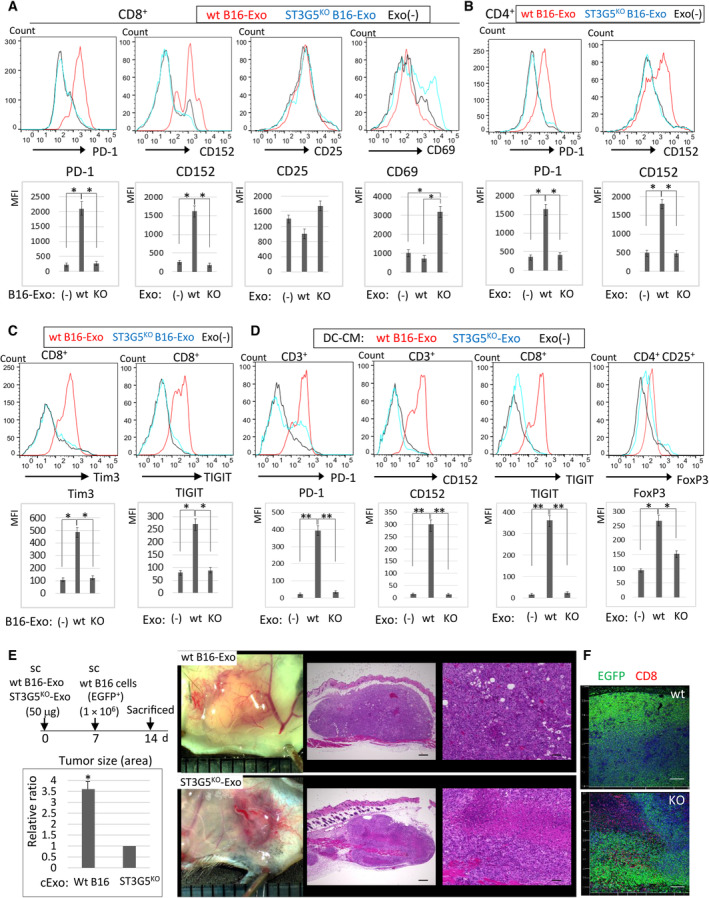
ST3G5^high^‐cExo increased immune checkpoint molecules and T‐cell exhaustion. (A, B) Immature DCs were treated by cExo (wt or ST3G5^KO^‐B16, 5 μg·mL^−1^) or left untreated, and further incubated with CD3^+^ T cells isolated from mouse spleen for 3 days. Viable cells were gated for CD8^+^ or CD4^+^ T cells and further characterized for expression of indicated molecules. In some experiments, the results were normalized by the cell number and expressed as unit area (U.A.). (C) Expression of exhaustion markers in CD8^+^ T cells. (D) Immature DCs were treated by wt or ST3G5^KO^ B16‐cExo (5 μg·mL^−1^) or left untreated. After washing out the exosomes, the conditioned medium (CM) was collected from these DCs, and added to CD3^+^ T cells for FACS analysis. (A–D) Bottom panels: Mean fluorescence intensity (MFI) of each samples (mean ± SD, five independent experiments). *P < 0.01, **P < 0.006, Student's *t*‐test. (E, F) Exosomes of wt or ST3G5^KO^‐B16 cells (50 μg) were s.c injected into 6‐week‐old C57BL/6 mice (*n* = 5, each group), and EGFP^+^ wt‐B16 cells (1 × 10^6^) were injected at the same site on Day 7. Mice were sacrificed on Day 14, and dissected tumors were fixed and the maximum cut surface was subjected to H&E staining, and tumor area was quantified (E, mean ± SD, *P < 0.01, Student's *t*‐test. *n* = 5), or immunofluorescence staining of CD8 (red) and EGFP (green) (F). Representative images are shown. Bar, 100 μm (E), 200 μm (F).

The effects of ST3G5^high^‐cExo on the immune cells, that is, DC and T cells were further examined. Murine iDCs were treated with exosomes from wt‐ or ST3G5^KO^ B16 cells, and further co‐cultured with splenic T cells for 3 days. When co‐culture with iDCs treated with wt B16‐cExo, PD‐1 and CD152 were induced in CD8^+^ T cells, which were higher than those co‐cultured with ST3G5^KO^ B16‐cExo‐pulsed iDCs (Fig. 5A). In addition, apoptosis of CD8^+^ T cells was also increased, whereas CD25^+^ and cell surface CD69 [38] (activated T‐cell markers) were not elevated in the co‐culture with wt B16‐cExo‐iDCs (Fig. 5A). Similar results were obtained for CD4^+^ T cells (Fig. 5B). We examined the cell cycle using flow cytometry and found that CD8^+^ T cells in the S phase were increased, whereas cells in G2 phase were reduced by the co‐culture with wt B16‐cExo‐iDC (Fig. S3B), suggesting disturbance of cell cycle progression.

We further examined CD8^+^ T‐cell exhaustion by co‐culture with cExo‐pulsed iDCs and detected an elevation in TIM‐3 and TIGIT by co‐culture with wt B16‐cExo‐iDCs (Fig. 5C).

However, the direct effects of cExos on T cells were not obvious. When wt‐ or ST3G5^KO^ B16‐cExos were added to CD3^+^ T cells, neither the expression of immune checkpoints nor apoptosis of T cells was elevated (Fig. S3C). In addition, when soluble GM3 was added to the culture medium of lymphocytes, apoptosis increased (Fig. S3D), whereas the number of CD4^+^ or CD8^+^ T cells and the levels of PD‐1 and CD152 (Fig. S3D) remained unchanged, indicating different responses from cExos.

We further examined whether the effects of cExo‐iDCs on T cells depended on the soluble factors released from DCs. When T cells were incubated with a CM of wt B16‐cExo‐treated iDCs, induction of PD‐1, CD152, and TIGIT was evident in T cells and CD25^+^ FoxP3^+^ Tregs were increased (Fig. 5D). In addition, lactate also increased in the CM of wt B16‐cExo‐iDCs (Fig. S3E). These events were attenuated by ST3G5^KO^‐B16‐cExo (Fig. 5D, Fig. S3E), suggesting that cytokines and/or lactate released from these DCs affect T‐cell functions.

The cytotoxic effects of T cells on cancer cells were further examined. When B16 cells, in which MyrPalm‐EGFP labeled the cell membrane, were added to the cExo‐pulsed iDCs and T cells, cleaved caspase‐3 was induced in cancer cells solely by the ST3G5^KO^ B16‐cExo‐iDC/T‐cell mixture (Fig. S4A‐c). A combination of wt B16‐cExo‐iDC/T cells did not induce cancer cell apoptosis, whereas apoptosis was prominent in lymphocytes (Fig. S4A‐b).

### 3.6 ST3G5^high^‐cExo induces immunosuppression in MS via upregulation of CCL5

The tumorigenicity of WT and ST3G5^KO^ B16 cells was examined *in vivo*. Subcutaneous injection of WT and ST3G5^KO^ B16 cells has no major effect on tumor size (Fig. S4B). Therefore, B16‐cExos were preinjected, and wt B16 cells were injected 7 days later, which is similar to the method for activation of immunoreaction by preinjection of dead cancer cells [39]. As a result, the tumors became larger in mice preinjected with wt B16‐cExo than ST3G5^KO^ B16‐cExo (Fig. 5E, Fig. S4C). Histologically, tumors preinjected with ST3G5^KO^ B16‐cExo contained many lymphocytes and neutrophils, with numerous dead cancer cells (Fig. 5E). Meanwhile, infiltration of CD25^+^CD8^+^ activated T cells was reduced by the preinjection of wt B16‐cExo (Fig. 5F).

**Fig. 6:**
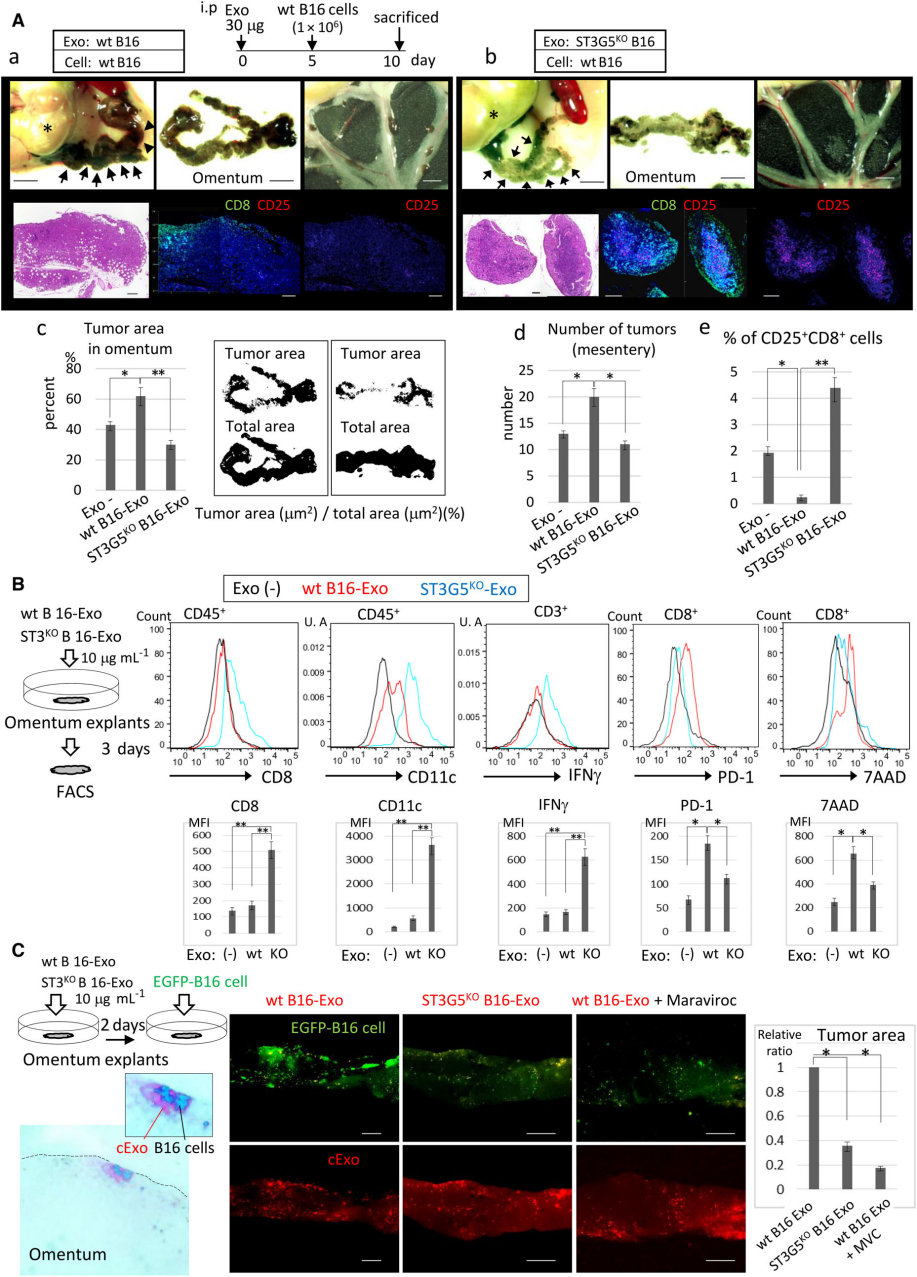
ST3G5^high^‐cExo caused immunosuppression in milky spots (MS) and accelerated omentum metastasis. (A) Exosomes of wt (a) or ST3G5^KO^‐B16 cells (b) (30 μg, each) were i.p injected into C57BL/6 mice (*n* = 5, each group), and wt B16 cells (1 × 10^6^) were i.p injected on Day 5. Mice were sacrificed on Day 10. (Control mice without preinjection of exosomes were shown in Fig. S4D.) Upper panels: Representative images of the omentum and mesentery. Arrows indicate the omentum. Tumors were detected by melanin in cancer cells (black). Asterisk: stomach. Bar, 2.5 mm. Lower panels: Omentums were fixed, paraffin‐embedded, and sectioned for H&E staining (left) and immunostaining with anti‐CD8 (green), anti‐CD25 (red), and DAPI. Merged image (middle) and CD25 alone (right). Bar, 200 μm. (c) Tumor area in omentum was quantified by imagej software (examples were shown in right) and expressed as percentage of tumor area in total area of omentum (*n* = 5, mean ± SD, *P < 0.05, **P < 0.01, Student's *t*‐test). (d) The number of tumors in mesentery was counted and analyzed the significance of the difference by Student's *t*‐test. Results are shown as means ± SD (*n* = 5, *P < 0.01, Student's *t*‐test). (e) The area of CD8^+^CD25^+^ T cells in omentum tumors was measured and expressed as the percentage of the tumor area in each specimen. Five tumors were analyzed individually (mean ± SD, *P < 0.01, **P < 0.006, Student's *t*‐test). (B) Omentums were excised from C57BL/6 mice and cultured in medium containing Wt or ST3G5^KO^‐B16‐cExo (10 μg·mL^−1^), or left untreated for 3 days. Omentums were dispersed and subjected to FACS as indicated. Mean ± SD, *P < 0.05, **P < 0.01, Student's *t*‐test. (C) Omentum explants were treated by ExoSparkler‐labeled cExo (red) as above. In some experiments, Maraviroc (10 μg·mL^−1^) was added together with the exosomes. EGFP^+^ B16 cells were added to these explants (5 × 10^5^ cells per well) and further incubated for 3 days. Representative appearance of the omentum. Left: Pseudo‐color image of the co‐localization of B16 cells and cExo on the explant. Tumor area was measured by quantification of green fluorescence (EGFP) and normalized to the cExo area (red) in the omentum. Bar, 1.5 mm. The results are shown by the relative ratio of the explant treated by wt B16‐cExo without inhibitor. Mean ± SD, *P < 0.01, Student's *t*‐test. (B, C) Five explants were analyzed in each group.

We analyzed cancer metastasis to the omentum using similar sequential injection of cExo and cancer cells. Metastasis of wt B16 cells was augmented by preinjection of wt B16‐cExo compared with ST3G5^KO^ B16‐cExo or without exosome preinjection (Fig. 6Aa–d, Fig. S4D). Histologically, infiltration of CD25^+^CD8^+^ T cells was significantly reduced in tumors preinjected with wt B16‐cExo (Fig. 6A‐a,b,e).

The immune microenvironment in the omentum was further examined. When the mouse omentum was excised and cultured, the addition of ST3G5^KO^ B16‐cExo increased the number of CD8^+^ T cells and CD11c^+^ DCs, accompanied by elevation of interferon (IFN)‐γ, which was less induced by wt B16‐cExo (Fig. 6B). Conversely, wt B16‐cExo elevated PD‐1 expression and apoptosis in CD8^+^ T cells (Fig. 6B). When these cExo‐treated explants were incubated with cancer cells, more cancer cells were attached to and survived in the wt B16‐cExo‐treated omentum (Fig. 6C).

We observed similar immunosuppressive effects of ST3G5^high^‐cExo by 44As3 cells. Elevation of PD‐1 and CD152 in CD8^+^ and CD4^+^ T cells was exacerbated, while activated CD8^+^ T cells (CD25^+^, CD69^+^ or IL‐2^+^) were not increased by iDCs pulsed with wt 44As3‐cExo (Fig. S5A,B). T‐cell exhaustion (detected by TIM‐3, TIGIT, and LAG3) was also enhanced by wt 44As3‐cExo‐iDCs (Fig. S5C), and similar immunosuppressive changes were induced in omentum explants by wt 44As3‐cExo (Fig. S5D).

To elucidate the common immunosuppression factors by ST3G5^high^‐cExo in cultured iDCs and omental MS, RNA sequence analysis was performed. Wt B16‐cExo significantly altered the gene‐expression profile of iDCs compared with ST3G5^KO^ B16‐cExo or untreated iDCs (Fig. S6A). Gene Ontology analysis revealed that genes classified as ‘cytokine/chemokine activity’ or ‘inflammation related’ were upregulated in iDCs by wt B16‐cExo (Fig. S6A), while ‘cell cycle’ or ‘cell division’ were downregulated. Similar RNA‐Seq was performed in omental MS with or without treatment with B16‐cExo (Fig. S6B). When the 100 most upregulated genes by wt B16‐cExo in DC and MS were compared, three common genes, CCL5, CCL22, and Arg1, were detected (Fig. S6C). Elevation of CCL5 expression was confirmed at the mRNA (Fig. S6D) and protein levels in iDCs treated with wt B16‐cExo and 44As3‐cExo (Fig. S6E).

When maraviroc was added to the co‐culture of wt B16 or 44As3‐cExo‐pulsed iDCs and T cells, the expression of PD‐1 and TIGIT was decreased in CD8^+^ T cells, while IFN‐γ was upregulated (Fig. S6F). Moreover, wt B16‐cExo‐mediated cancer cell metastasis was blocked following the omentum explants treatment with maraviroc (Fig. 6C) and partially suppressed by anti‐mouse PD‐1 antibody (Fig. S7A).

## 4 Discussion

In this study, we observed that ST3G5^high^ cancer cells secreted exosomes containing HIF1α and glycolytic enzymes. The uptake of these exosomes upregulated CD169, a cell surface receptor of GM3 in MΦs and DCs, which further promoted the incorporation of exosomes via positive‐feedback loop. ST3G5^high^‐cExo‐mediated upregulation of CD169 via HIF1α is consistent with that CD169 is induced in bone marrow MΦs under the hypoxic condition [40]. Recipient MΦs expressed inflammatory cytokines via activation of NF‐κB, and induced CAF transformation of mesothelial cells through MMT. ST3G5^high^‐cExo also upregulated immune checkpoints and induced exhaustion and apoptosis in T cells. These effects were substantially dependent on CCL5 secreted by cExo‐uptake DC and MΦs. Cancer‐associated fibroblast transformation of mesothelial cells and T‐cell suppression were observed in MS of the omentum, which facilitated the premetastatic niche for ST3G5^high^ cancer cells (Fig. S7B).

We prepared exosomes from cancer cells cultured in the hypoxic condition. In the initial comparison of the exosomes, higher amounts of HIF1α and LDHA were isolated in the absence of serum (Fig. S7C). Since the appearance of cancer cells was not apoptotic in the culture, and exosomes did not contain apoptotic vesicles judged by cleaved caspase 3 (Fig. S7C), we isolated them under serum‐starved hypoxic conditions.

ST3G5‐synthesized a‐series gangliosides inhibit transforming growth factor‐β (TGF‐β) signaling‐induced EMT [41]. These gangliosides promote lipid raft localization of TGF‐β type I receptor (TβRI), thereby inducing TβRI ubiquitination and degradation [41]. The direct effect of cExo on PMC was relatively weak, whereas MMT was strongly induced by co‐culture with cExo‐MΦs. Although ST3G5 and GM3 were contained in 44As3‐cExo, the concentration of ST3G5 was not high in the exosomes (Fig. S7D), and neither ST3G5 expression nor GM3 levels were upregulated in the recipient MΦs or co‐cultured PMCs (data not shown). As the primary reason for MMT by these MΦs, we observed enhanced inflammatory signaling, including the upregulation of CCL5 in MΦs. MMT decreases a mesothelial barrier and accelerates co‐invasion of PMC‐derived CAFs and cancer cells [22], which activate peritoneal metastasis.

CC‐chemokine ligand 5 induces EMT in various tissues [37], which partially depends on the transcriptional activation of STAT3 [42]. Activated STAT3 signaling upregulates EMT regulators, for example, ZEB1/2, Snail, and Slug, leading to MMT [43]. Our results suggest CCL5‐STAT3 signaling as a promising target for preventing peritoneal dissemination mediated by ST3G5^high^‐cExo. In addition to CCL5, CCL2, and TIMP‐1 were also elevated in the CM of MΦs treated by ST3G5^high^‐cExo. It is known that tumor‐associated MΦs secrete CCL2, which promotes EMT in cancer cells [44]. Also, TIMP‐1 is known as a typical fibrosis‐related molecule and induces αSMA in fibroblasts [45].

The expression of immune checkpoints and exhaustion of T cells is caused by excessive or sustained T‐cell receptor‐mediated T‐cell activation. Besides, inflammatory cytokines, including IL‐2, IL‐6, IL‐7, IL‐15, IL‐17, and CXCL7, induce the expression of PD‐1, TIM3, and LAG3 [46]. Furthermore, lactate, produced by glycolytic metabolism in the tumor microenvironment, also induces PD‐1 expression in T cells [47]. Induction of immune checkpoints and T‐cell exhaustion by the CM of DCs treated with ST3G5^high^‐cExo supported that cExo‐mediated secretion of inflammatory cytokines and lactate caused these events. By contrast, GM3 did not seem to contribute to T‐cell suppression as it was not elevated in cExo‐treated MΦs and DCs, while treatment with GM3 did not show strong effects on T cells.

Although MMT‐mediated peritoneal fibrosis by ST3G5^high^‐cExo might have suppressed immunoreactions by preventing immune cell infiltration, addition of anti‐PD‐1 antibody at least partially ameliorated the peritoneal metastasis in our mouse model. Therefore, ST3G5^high^‐cExo contributed to the premetastatic niche formation by both MMT of PMC and T‐cell suppression. In addition, DC maturation, which indicates a change from phagocytic DC to mature antigen‐presenting cells with high expression of major histocompatibility complex (MHC) molecules [48], is caused by the uptake of some exosomes [49]. However, I‐A/I‐E (MHC II) expression was weaker in iDCs treated with ST3G5^high^‐cExo compared with those treated with ST3G5^KO^‐cExo (Fig. S7E), which might have attenuated T‐cell activation.

GM3‐containing liposomes are incorporated by CD169^+^ MΦs, which transfer target antigens to DCs, thereby inducing robust antigen‐specific CD4^+^ or CD8 T^+^ cell response [50, 51]. Our observation that exosomes secreted from ST3G5^high^ cancer cells attenuated DC and subsequent T‐cell activation suggests that ST3G5 expression in cancer cells may both activate and suppress anti‐cancer immune responses based on the contents in their exosomes. This may explain the dual correlations of ST3G5 with poor and better prognosis [52, 53, 54, 55]. Although ST3G5 in cancer cells accelerated the incorporation of exosomes in MΦs, whether ST3G5 affects the delivery of exosomes remains to be determined. In future studies, we intend to investigate whether similar amount of exosomes from ST3G5^high^ and ST3G5^KO^ cancer cells arrive to MS via orthotopic implantation of mouse gastric cancer cells in the stomach.

Although the mechanism is nebulous, ST3G5^high^‐cExo contained higher amounts of HIF1α than ST3G5^KO^‐cExo, which activates NF‐κB and its downstream molecules, including CCL5 and PD‐L1 in MΦs and/or DC. CCL5 contributes to immune suppressive environment [56], and MΦ‐derived CCL5 facilitates the immune escape of colorectal cancer cells via the STAT3‐PD‐L1 pathway [57]. Furthermore, induction of PD‐L1 in MΦs decreases the production of IFN‐γ in co‐cultured CD8^+^ T cells [35]. In clear cell renal cell carcinoma (CCRCC), both an increase in CCL5^+^ MΦs and a positive correlation between ST3G5 expression and poor survival have been reported [14, 37]. Therefore, suppressive immune microenvironment and ST3G5 and CCL5 expression in gastric cancer and other tumors should be examined in future studies.

Our immunohistochemical analysis in human gastric cancers showed that ST3G5 expression was observed at high incidence in the primary tumors, which caused peritoneal relapse (46%, 6/13 cases), compared with those without peritoneal relapse (9%, 1/11 cases; Table 2, Fig. S8A). Notably, ST3G5 expression was heterogeneous within the same tumor, and ST3G5^high^ cancer cells were detected in the invasion front in some cases (3/6 cases), although it was not exclusive to the scirrhous‐type cancer (Table 2, Fig. S8Aa–c). ST3G5^high^ cancer cells were also detected in disseminated tumor nodules in the peritoneum (Fig. S8A, bottom). Further study with larger scale will be necessary to show the statistically significant differences. Analysis of RNA sequencing dataset in multi‐omics profiles of gastric cancer cells purified from malignant ascites [58] using GEO2R and rnaseqchef software [59] showed that the mRNA of genes including HIF1α, CCL5, and EMT‐related transcription factor SNAI2 (Slug) were upregulated in ST3G5^high^ cancer cells compared with ST3G5^low^ cancer cells (Fig. S8B,C). These observations suggest that ST3G5^high^ cancer cells may activate similar pathways in premetastatic niche via exosomes.

## 5 Conclusions

Exosomes secreted from ST3G5^high^ cancer cells contribute to two major events in the premetastatic niche in MS, namely induction of CAF transformation of PMCs and immunosuppression. These events largely depended on the secretion of CCL5 from the exosome‐incorporated MΦs and DCs. CCL5‐STAT3 pathway upregulated transcription factors necessary for MMT (EMT), and induced immune checkpoint molecules and exhaustion in T cells. The CCR5 antagonist Maraviroc attenuated peritoneal dissemination promoted by ST3G5^high^ cancer cell exosomes. Our results suggest ST3G5 as a therapeutic target for preventing premetastatic niche of peritoneal dissemination.

## Conflict of interest

The authors declare no conflict of interest.

## Author contributions

MT contributed to the conceptualization, writing—original draft, and writing—review and editing. MH, KT, GI, SK, MU, and MT contributed to the experiments and investigation. KY and MY contributed to the methodology (material support). KT, GI, SK, AG, JA, and MT contributed to the analysis and interpretation of data.

## Supporting information


**Fig. S1.** Expression of ST3G5 in cancer cell lines.
**Fig. S2.** Effects of cExo on MΦs and PMCs.
**Fig. S3.** Direct effects of ST3G5^high^‐cExo on T cells.
**Fig. S4.** ST3G5^high^‐cExo attenuates T‐cell‐mediated cancer cell cytotoxicity.
**Fig. S5.** ST3G5^high^ 44As3‐cExo increased immune checkpoint molecules and T‐cell exhaustion.
**Fig. S6.** RNA‐Seq analysis of cExo‐treated iDC and MS.
**Fig. S7.** Model of ST3G5^high^‐cExo‐mediated premetastatic niche.
**Fig. S8.** ST3G5 expression in human gastric cancer specimens.

## Data Availability

The datasets used and/or analyzed during the current study are available from the corresponding author upon reasonable request.
