# Supplementary material for: Exosomes secreted by ST3GAL5 high cancer cells promote peritoneal dissemination by establishing a premetastatic microenvironment
Source: Mol Oncol. 2023 Sep 27;18(1):21–43. doi: 10.1002/1878-0261.13524 (PMC10766203; doi:10.1002/1878-0261.13524)

Supplementary figure legends

Fig S1. **Expression of ST3G5 in cancer cell lines.** (A) Schematic diagram of major GSL pathways, including ganglioside synthesis glycosyltransferases. (B) Cell lysates from various cancer cell lines and CAF of human scirrhous gastric cancer (CAF-37) were subjected for immunoblot analysis by anti-ST3G5 antibody. (C) GD3 and GD2 in the wild-type (wt) 44As3 cells were evaluated by flow-cytometer. SK-MEL-28 cell and U87MG cells were used as the control.

Fig S2. **Effects of cExo on MΦs and PMCs.** (A) 44As3 cells (wt or ST3G5^{KO} clones; 1×10^6) were injected subcutaneously into 6-week-old nude mice, and sacrificed at day 10. Representative appearance of the tumors. Tumor size was measured by caliper at the time of sacrificed, and shown by the relative ratio to the tumors of wt 44As3 cells. Five mice were examined in each group. **P* < 0.01. (B) The expression level of indicated molecules in 44As3 cells (upper) or B16 cells (bottom) in Fig 1H and Fig 1I. The results were summarized from three independent experiments, and shown by the relative ratio to the wild-type cells under the standard condition. *P < 0.01. (C) Cytokine array analysis was performed as described in Materials and Methods using CM collected from MΦs treated with wt or ST3G5^{KO} 44As3-cExo (5 μg/mL) or untreated MΦs. Array images are shown. Right: Array images were quantified as described in Materials and Methods, and expressed as the mean pixel density. The results from three independent experiments are shown as the mean ± SD. *P < 0.01. (D, E) MΦs treated with cExo of wt or ST3G5^{KO}-44As3 cells (5 μg/mL) were subjected for Western blot analysis of the glycolysis related molecules (D), or FACS analysis of PD-L1 expression (E, three independent experiments. **P* < 0.01). (F) MΦs were treated by exosomes of B16 cells (wt or ST3G5^{KO} clone) (5 μg/mL) for two days, and the cells were subjected for immunoblot analysis. (G, H) MΦs were incubated with exosomes as above (G: wt or ST3G5^{OE} 44PE-cExo, H: wt 44As3-cExo) for 2 days, and mixed with the biotin-labeled PMCs (1:1 ratio) and co-cultured for 5 days. Cells were collected and biotin$^+$/CD11b$^-$/F4/80$^-$ PMCs were selected using mag-beads columns (Materials and Methods), and subjected for immunoblot analysis. In H, Maraviroc (1 μM) was added to the medium during the coculture of MΦ and PMC. (D, F, G, H) The intensities of each band were quantified, and normalized by β-actin or α-tubulin. Expression of phosphorylated protein was further adjusted by each total protein. The relative ratios were described below the panel.

Fig S3. **The direct effects of ST3G5^{high}-cExo on T cells.** (A) Representative images of

the exosome-negative control nude mice injected with DiI-labeled wt 44As3 cells as described in Fig 3A. Asterisk: bladder, S: stomach. Bar, 2.5 mm. (B) Immature DCs were treated by cExo (wt or ST3G5^{KO}-B16, 5 μg/mL) or untreated, and further incubated with CD8^{+} T cells isolated from mouse spleen for 2 days. CD8^{+} T cells were collected by mag-beads columns, and subjected to flow cytometry analysis to investigate cell cycle status as described in Supplementary materials and methods. (C) Exosomes of wt or ST3G5^{KO} B16 cells were directly added to CD3^{+} T cells (5 μg/mL), and subjected for FACS analysis of PD-1, CD152, and 7AAD. (D) Mouse spleen lymphocytes were incubated with or without soluble GM3 (20 μM) for 3 days, and subjected for FACS analysis as indicated. Arrow indicated the increase of CD8^{+} T cell apoptosis by GM3. (E) Conditioned medium of immature DCs treated by wt or ST3G5^{KO} B16-cExo as in Fig 5D was collected, and quantified lactate by ELISA as described in Supplementary Materials and Methods. *$P < 0.01$.

Fig S4. **ST3G5^{high}-cExo attenuate T cell mediated cancer cell cytotoxicity.** (A) Immature DCs were untreated (a), treated by wt (b) or ST3G5^{KO} (c) B16-cExo, and incubated with CD3^{+} T cells. B16 cells expressing MyrPalm-EGFP, which labeled cell membrane were added to these co-culture, and further incubated for 24hs. The cells were immunostained with anti-cleaved caspase 3 antibody and anti-CD3 antibody. Insets (bottom): cleaved caspase 3 (red), CD3^{+} T cells (white). (c) Arrows indicate the staining of cleaved caspase-3 in cancer cells. The image of the same field was shown in the bottom. Asterisks indicate the positions of nucleus. Bar, 50 μm (a, b) or 10 μm (a, b: insets, c). *$P < 0.01$. Random 100 cancer cells were analyzed. (B) Wt or ST3G5^{KO} B16 cells (1x10^{6}) were s.c injected in C57BL/6 mice. Tumors were resected at day7, fixed and subjected to H&E staining. Right: Tumor size was compared by the area (mm^{2}) of the maximum cut surface, and expressed as the relative ratio. Bar, 200 μm. Five mice were analyzed in each group. (C) Examples of quantitation of the tumor area using the samples obtained in Fig 5E experiment. The areas of tumors were quantified by ImageJ software. Bar, 200 μm. (D) Control mice of Fig. 6A. Mice were i.p injected wt B16 cells (1x10^{6}) without pre-injection of exosomes. Representative images of omentum and mesentery (bar, 2.5 mm). H&E staining of the omentum (bar, 200 μm). Right: Examples of quantification of the tumor area.

Fig S5. **ST3G5^{high} 44As3-cExo increased immune checkpoint molecules and T cell exhaustion.** (A-C) Immature DCs were treated by cExo (wt or ST3G5^{KO}-44As3, 5 μg/mL) or left untreated, and further incubated with CD3^{+} T cells isolated from

mouse spleen for 3 days. Viable cells were gated for $CD8^+$ (A) or $CD4^+$ (B) T cells, and further characterized for expression of indicated molecules. (C) Expression of exhaustion markers in $CD8^+$ T cells. (A-C, bottom) Results of five independent experiments. *$P < 0.01$. (D) Omentums were excised from C57BL/6 mice and cultured. Omentum explants were incubated with wt or $ST3G5^{KO1}$-44As3-cExo (10 μg/mL) or left untreated as described in Fig 6B. Explants were dispersed, and subjected for FACS as indicated. *$P < 0.05$, **$P < 0.01$. Five explants were analyzed in each group.

Fig S6. **RNA-seq analysis of cExo-treated iDC and MS.** (A) Microarray analysis was performed using mRNA of iDCs treated with wt or $ST3G5^{KO}$ B16-cExo, and control untreated iDCs (cExo(-)). Left: Gene set enrichment analysis of up (> 4.0 fold) or down regulated (< 4.0 fold) genes in iDCs treated by wt B16-cExo compared with iDCs treated by $ST3G5^{KO}$ B16-cExo. Right: Heatmap of differentially expressed gene (DEG). (B) RNA-sequence analysis of omentum MS treated by cExo. Exosomes of wt B16 cells (30 μg) were i.p injected in $WT1^{CreERT2}$-tdT, and the MS were collected at day 3. Scatter plot of expression level between two groups. X-axis is control MS from untreated mice, and Y-axis is the value of MS in wt B16-cExo injected mice. Right: Heatmap of differentially expressed gene (DEG) list between two groups as indicated bottom. (C) Venn diagram of up-regulated genes in wt B16-cExo treated iDC (left), and wt B16-cExo treated omentum MC (right). (D, E) Immature DCs were treated by cExo of 44As3 cells or B16 cells (5 μg/ mL) as indicated, and subjected for qRT-PCR (D) or immunoblot analysis (E). *$P < 0.01$, **$P < 0.006$. (F) Maraviroc canceled the effects of $ST3G5^{high}$-cExo on T cells. $CD3^+$ T cells were cocultured with iDCs treated by wt B16 or 44As3-cExo as in Fig 5A-C, together with or without Maraviroc (MVC, 1 μg/mL) for 3 days. The cells were collected and examined expression of PD-1, TIGIT and IFN-γ in T cells by FACS analysis. Bottom; Results of three independent experiments. *$P < 0.01$.

Fig S7. **Model of $ST3G5^{high}$-cExo mediated pre-metastatic niche.** (A) Omentum explants were treated by ExoSparkler-labeled cExo (red, 10 μg/mL) or left untreated for 3 days. $EGFP^+$ B16 cells were added to these explants ($5x10^5$ cells/well) with anti-mouse PD-1 antibody or control rat IgG2a (1 μg/mL), and further incubated for 3 days. Representative appearance of the omentum. Bar, 1.5 mm. Tumor area was measured by quantification of green fluorescence (EGFP), and the results are shown by the relative ratio to the explant treated by wt B16-cExo with control IgG. Five explants were analyzed in each group. *$P < 0.01$. (B) Exosomes of $ST3G5^{high}$ cancer

cells contained HIF1α and glycolytic enzyme, which upregulated CD169 in MΦs and DCs, leading to increase incorporation of $GM3^{+}$ exosomes. The recipient MΦs and DCs turned to express inflammatory cytokines including CCL5 and lactate, which induced CAF transformation of mesothelial cells by MMT through activation of STAT3 pathway. $ST3G5^{high}$-cExo also suppressed T cells by upregulating immune checkpoint molecules and exhaustion, leading to establish the pre-metastatic niche in MS. Maraviroc (CCR5 antagonist) attenuated MMT and T cell suppression mediated by $ST3G5^{high}$-cExo. Upregulated molecules by $ST3G5^{high}$-cExo are depicted in red, and down-regulated molecules are depicted in blue. (C) 44As3 cells and B16 cells were cultured in medium containing 10% exosome-deprived FBS or without FBS under the hypoxic condition for 48 h. Exosomes were purified from the medium as described in Materials and Methods, and subjected for the immunoblot analysis. (D) Lysates were prepared from 44As3 cells and B16 cells cultured under the standard condition, or the exosomes isolated as described in Materials and Methods. Lysates (2.5 μg protein, each) were subjected for immunoblot analysis of ST3G5. Membranes were stained by Ponceau-S to evaluate the amount of loaded proteins. The band intensities were quantified, and the results were shown below the panels by the relative ratio to the cell lysate. (E) Immature DCs were prepared from bone-marrow cells, and treated by wt or $ST3G5^{KO}$ B16-cExo (5 μg/mL) for 2 days. Maturation of $CD11c^{+}$ DCs was assessed by I-A/I-E and/or CD86. Bottom: Results of three independent experiments. *$P < 0.01$. Right: The number indicates the percentage of I-A/I-E ^{high} DCs.

Fig S8. **ST3G5 expression in human gastric cancer specimens.** (A) Specimens of gastric cancers (total 24 cases; Summary was in Table 1) were immunostained with rabbit anti-ST3G5 antibody. Representative images of normal mucosa, primary tumor (case #1, #2; Bar, 200 μm. Insets, 50 μm.) and disseminated tumor (case #3; Bar, left 200 μm, right 50 μm.) were shown. The insets a-c in the right are the enlarged images of the boxed areas in case #2. Invasion front was shown in inset c. N; normal mucosa. (B, C) Analyze of RNA sequencing dataset GSE162214 in Gene Expression Omnibus (GEO). ST3G5 gene expression was examined in GSE162214 by GEO2R software (NCBI), and the highest 5 and lowest 5 samples were selected. The counts of gene expression profiles of those selected datasets were further analyzed by RNAseqChef web-based transcriptome analysis. (B) Heat map of genes showing differential expression in $ST3G5^{high}$ group and $ST3G5^{low}$ group. (C) Expression level of each genes are plotted as bar graph.

Fig S1

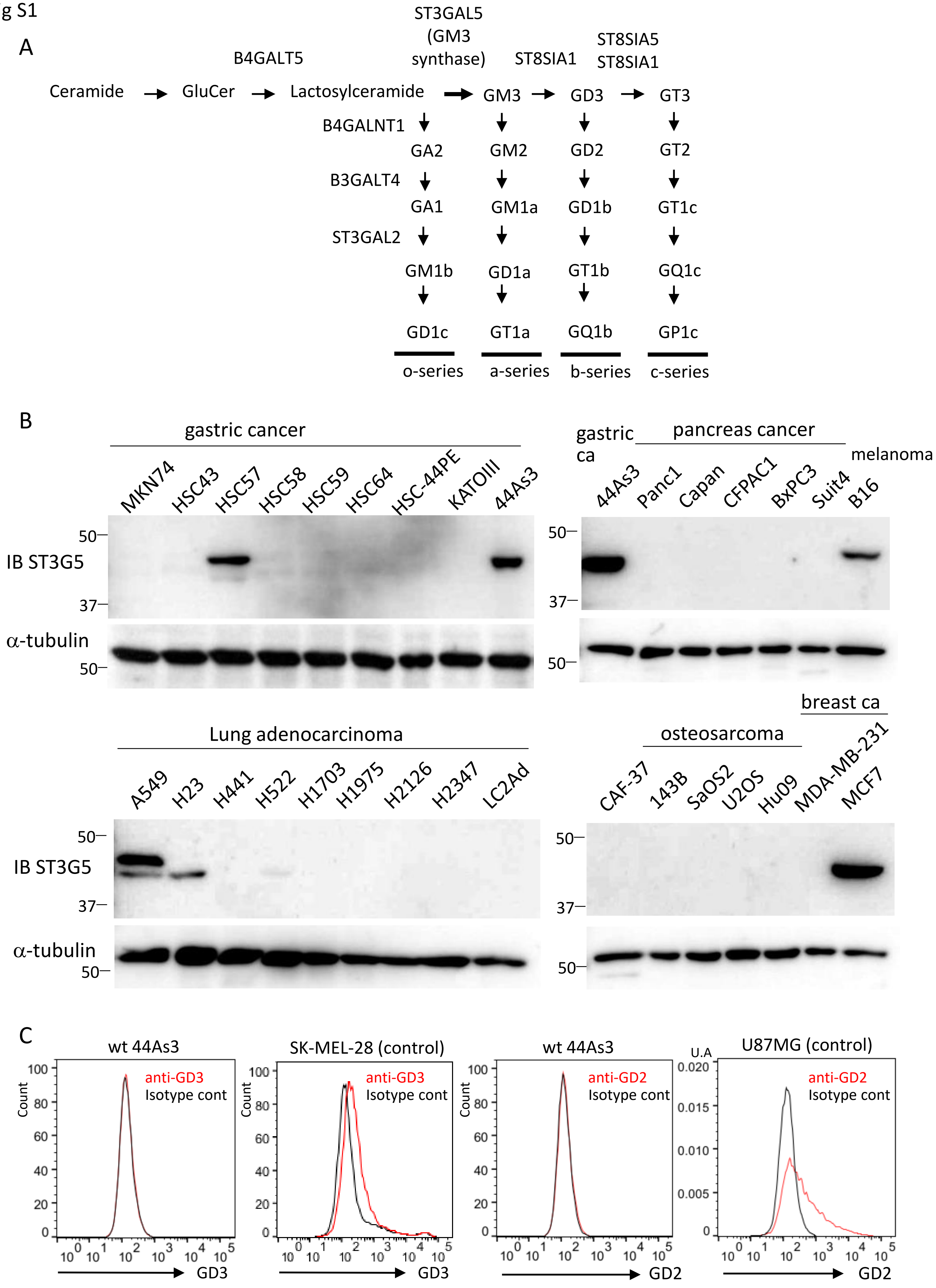
A
Ceramide
GluCer
B4GALT5
Lactosylceramide
ST3GAL5 (GM3 synthase)
GM3
ST8SIA1
GD3
ST8SIA5 ST8SIA1
GT3
B4GALNT1
GA2
GM2
GD2
GT2
B3GALT4
GA1
GM1a
GD1b
GT1c
ST3GAL2
GM1b
GD1a
GT1b
GQ1c
GD1c
GT1a
GQ1b
GP1c
o-series
a-series
b-series
c-series
B
gastric cancer
MKN74
HSC43
HSC57
HSC58
HSC59
HSC64
HSC-44PE
KATOIII
44As3
gastric ca
pancreas cancer
melanoma
Panc1
Capan
CFPAC1
BxPC3
Suit4
B16
IB ST3G5
α-tubulin
50
37
Lung adenocarcinoma
A549
H23
H441
H522
H1703
H1975
H2126
H2347
LC2Ad
osteosarcoma
breast ca
CAF-37
143B
SaOS2
U2OS
Hu09
MDA-MB-231
MCF7
C
wt 44As3
SK-MEL-28 (control)
U87MG (control)
anti-GD3
anti-GD2
Isotype cont
Count
U.A
GD3
GD2

Fig S2

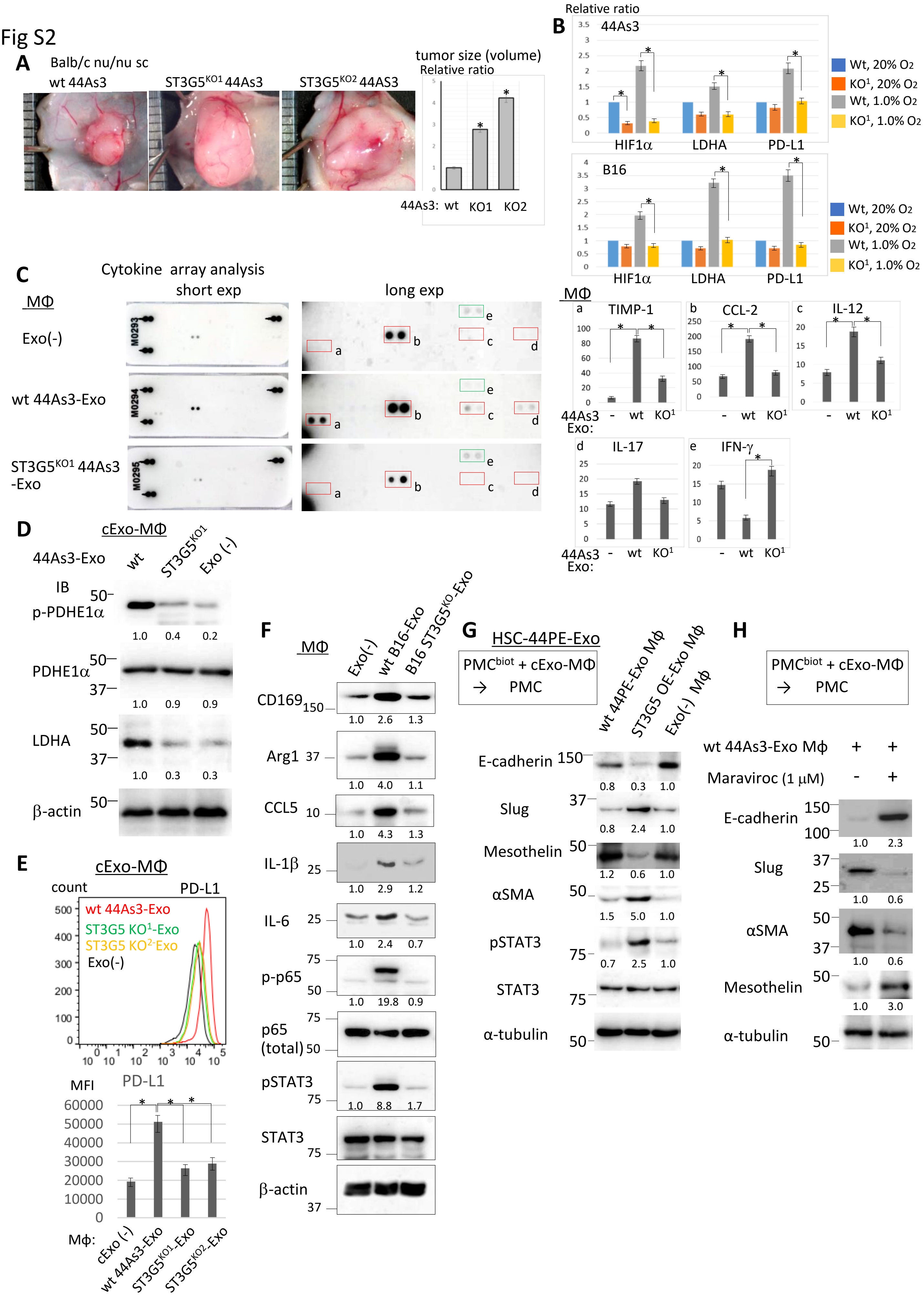
A
Balb/c nu/nu sc
wt 44As3
ST3G5KO1 44As3
ST3G5KO2 44AS3
tumor size (volume)
Relative ratio
44As3: wt KO1 KO2
B
Relative ratio
44As3
HIF1α LDHA PD-L1
Wt, 20% O2
KO1, 20% O2
Wt, 1.0% O2
KO1, 1.0% O2
B16
C
Cytokine array analysis
short exp
long exp
MΦ
Exo(-)
wt 44As3-Exo
ST3G5KO1 44As3 -Exo
a TIMP-1
b CCL-2
c IL-12
d IL-17
e IFN-γ
44As3 Exo: - wt KO1
D
cExo-MΦ
44As3-Exo wt ST3G5KO1 Exo (-)
IB
p-PDHE1α 1.0 0.4 0.2
PDHE1α 1.0 0.9 0.9
LDHA 1.0 0.3 0.3
β-actin
E
cExo-MΦ
count PD-L1
wt 44As3-Exo
ST3G5 KO1-Exo
ST3G5 KO2-Exo
Exo(-)
MFI PD-L1
Mϕ: cExo (-) wt 44As3-Exo ST3G5KO1-Exo ST3G5KO2-Exo
F
MΦ
Exo(-) wt B16-Exo B16 ST3G5KO-Exo
CD169 1.0 2.6 1.3
Arg1 1.0 4.0 1.1
CCL5 1.0 4.3 1.3
IL-1β 1.0 2.9 1.2
IL-6 1.0 2.4 0.7
p-p65 1.0 19.8 0.9
p65 (total)
pSTAT3 1.0 8.8 1.7
STAT3
β-actin
G
HSC-44PE-Exo
PMCbiot + cExo-MΦ → PMC
wt 44PE-Exo MΦ ST3G5 OE-Exo MΦ Exo(-) MΦ
E-cadherin 0.8 0.3 1.0
Slug 0.8 2.4 1.0
Mesothelin 1.2 0.6 1.0
αSMA 1.5 5.0 1.0
pSTAT3 0.7 2.5 1.0
STAT3
α-tubulin
H
PMCbiot + cExo-MΦ → PMC
wt 44As3-Exo Mϕ + +
Maraviroc (1 μM) - +
E-cadherin 1.0 2.3
Slug 1.0 0.6
αSMA 1.0 0.6
Mesothelin 1.0 3.0
α-tubulin

Fig S3

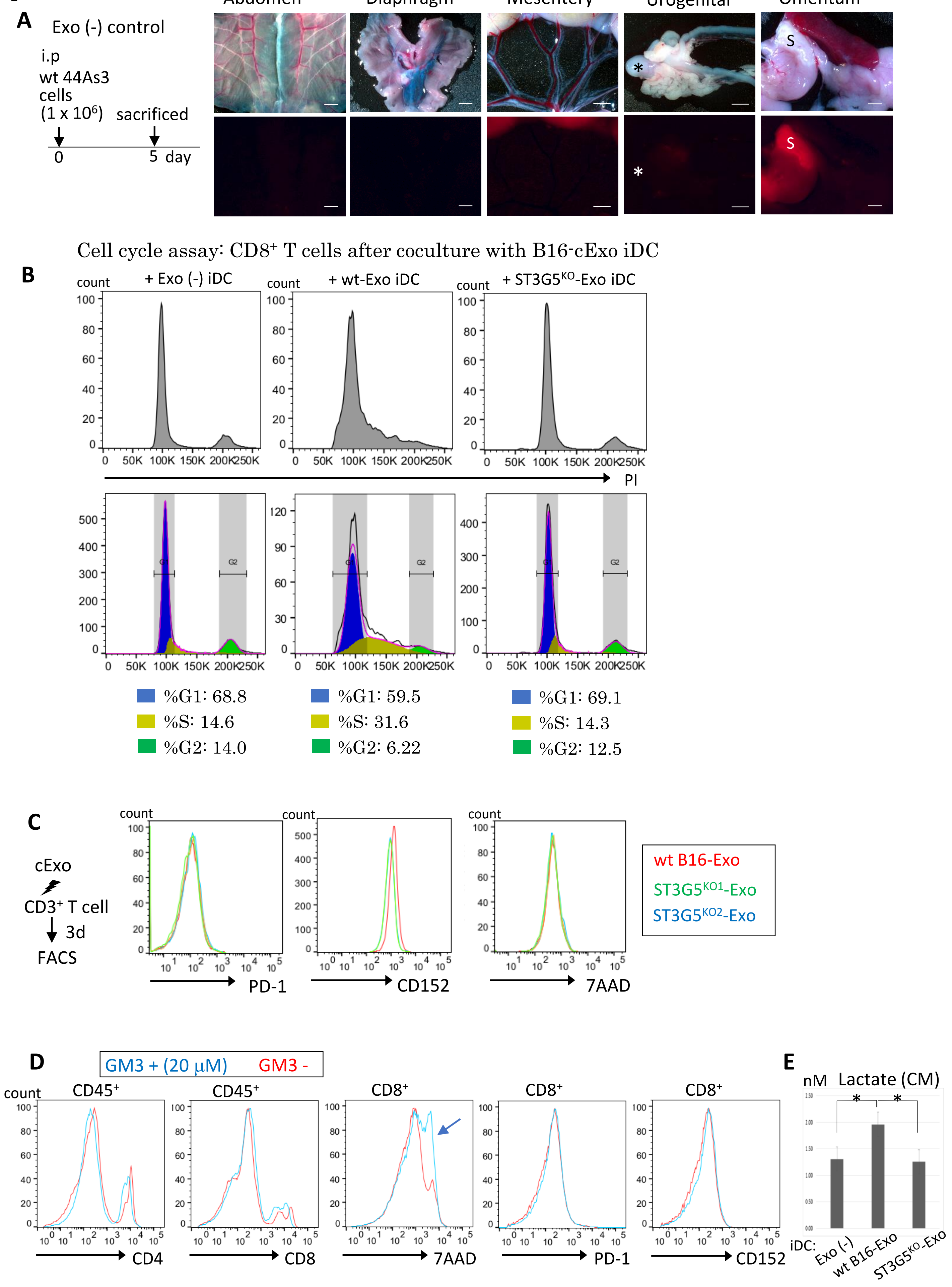

A
Exo (-) control
i.p
wt 44As3 cells
(1 x 10^6)
sacrificed
0
5 day
Abdomen
Diaphragm
Mesentery
Urogenital
Omentum
S
*
Cell cycle assay: CD8+ T cells after coculture with B16-cExo iDC
B
count
+ Exo (-) iDC
+ wt-Exo iDC
+ ST3G5KO-Exo iDC
PI
%G1: 68.8
%S: 14.6
%G2: 14.0
%G1: 59.5
%S: 31.6
%G2: 6.22
%G1: 69.1
%S: 14.3
%G2: 12.5
C
cExo
CD3+ T cell
3d
FACS
PD-1
CD152
7AAD
wt B16-Exo
ST3G5KO1-Exo
ST3G5KO2-Exo
D
GM3 + (20 μM)
GM3 -
CD45+
CD8+
CD4
CD8
7AAD
PD-1
CD152
E
nM
Lactate (CM)
iDC:
Exo (-)
wt B16-Exo
ST3G5KO-Exo

Fig S4

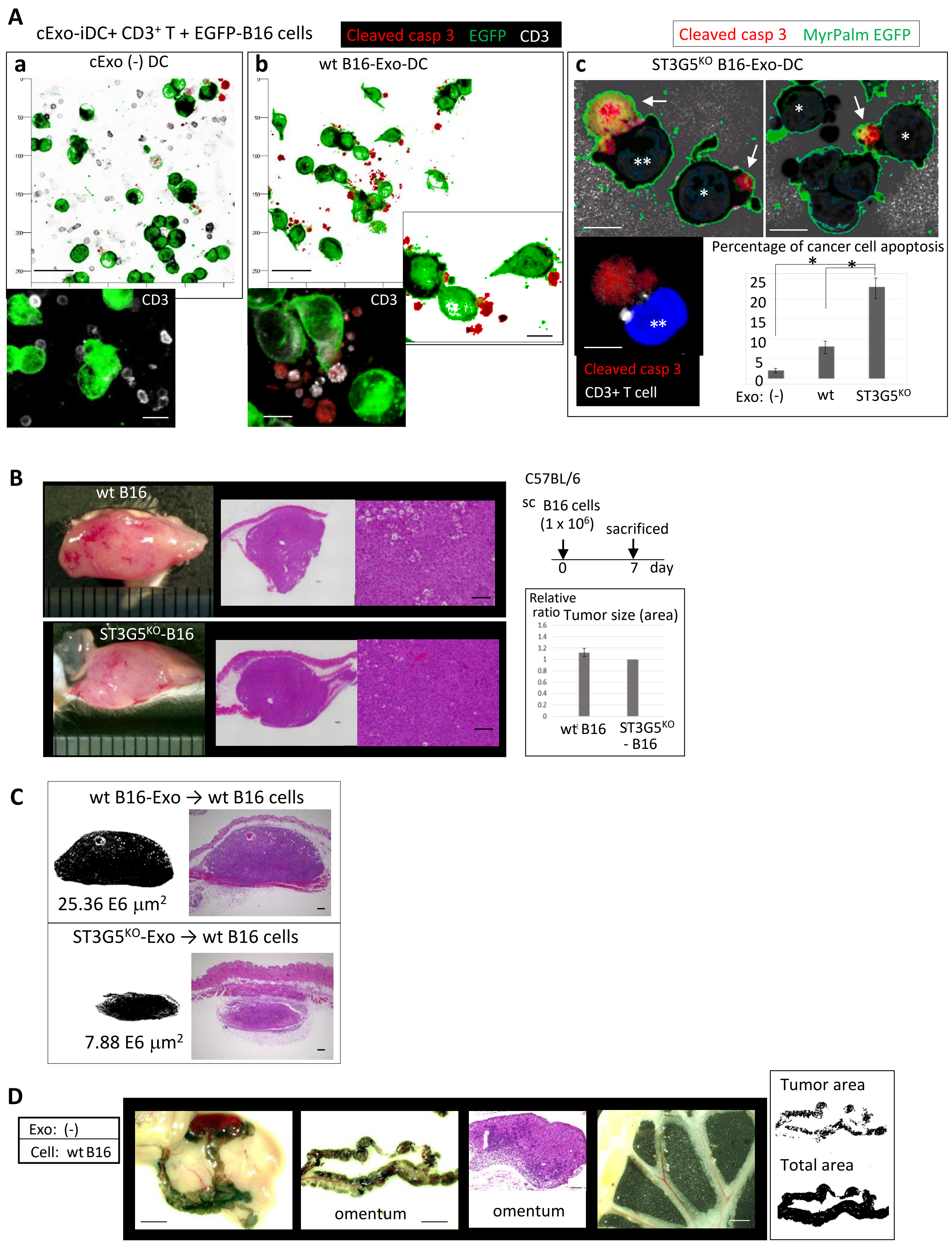
A
cExo-iDC+ CD3+ T + EGFP-B16 cells
Cleaved casp 3 EGFP CD3
Cleaved casp 3 MyrPalm EGFP
a
cExo (-) DC
CD3
b
wt B16-Exo-DC
CD3
c
ST3G5KO B16-Exo-DC
Percentage of cancer cell apoptosis
Cleaved casp 3
CD3+ T cell
Exo: (-) wt ST3G5KO
B
wt B16
ST3G5KO-B16
C57BL/6
sc B16 cells (1 x 10^6)
sacrificed
0 7 day
Relative ratio Tumor size (area)
wt B16 ST3G5KO - B16
C
wt B16-Exo → wt B16 cells
25.36 E6 μm²
ST3G5KO-Exo → wt B16 cells
7.88 E6 μm²
D
Exo: (-)
Cell: wt B16
omentum
omentum
Tumor area
Total area

Fig S5

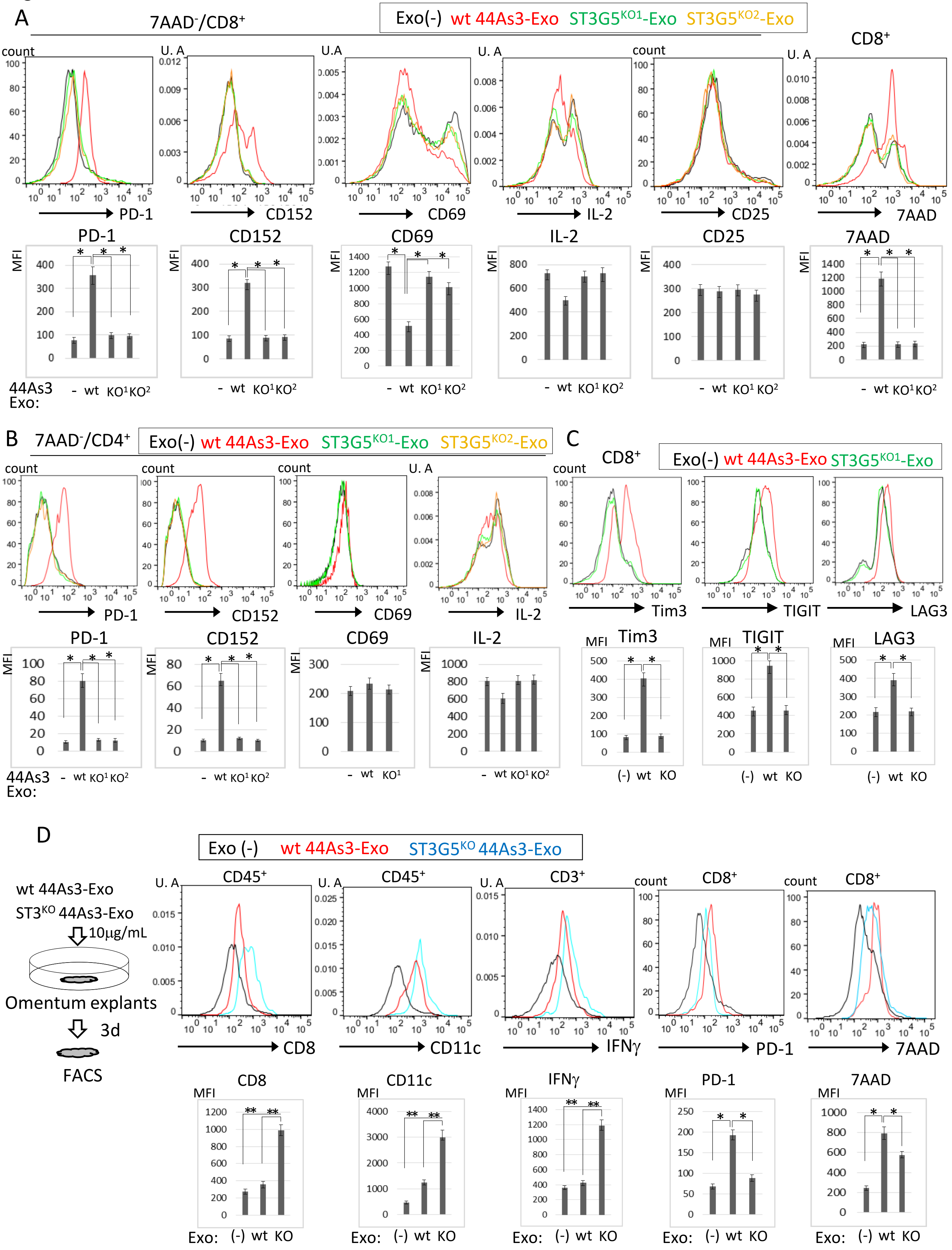

A
7AAD-/CD8+
Exo(-) wt 44As3-Exo ST3G5KO1-Exo ST3G5KO2-Exo
CD8+
PD-1
CD152
CD69
IL-2
CD25
7AAD
MFI
44As3 Exo: - wt KO1 KO2
B
7AAD-/CD4+
Exo(-) wt 44As3-Exo ST3G5KO1-Exo ST3G5KO2-Exo
C
CD8+
Exo(-) wt 44As3-Exo ST3G5KO1-Exo
Tim3
TIGIT
LAG3
(-) wt KO
D
Exo (-) wt 44As3-Exo ST3G5KO 44As3-Exo
wt 44As3-Exo
ST3KO 44As3-Exo
10µg/mL
Omentum explants
3d
FACS
CD45+
CD3+
CD8
CD11c
IFNγ
Exo: (-) wt KO

Fig S6

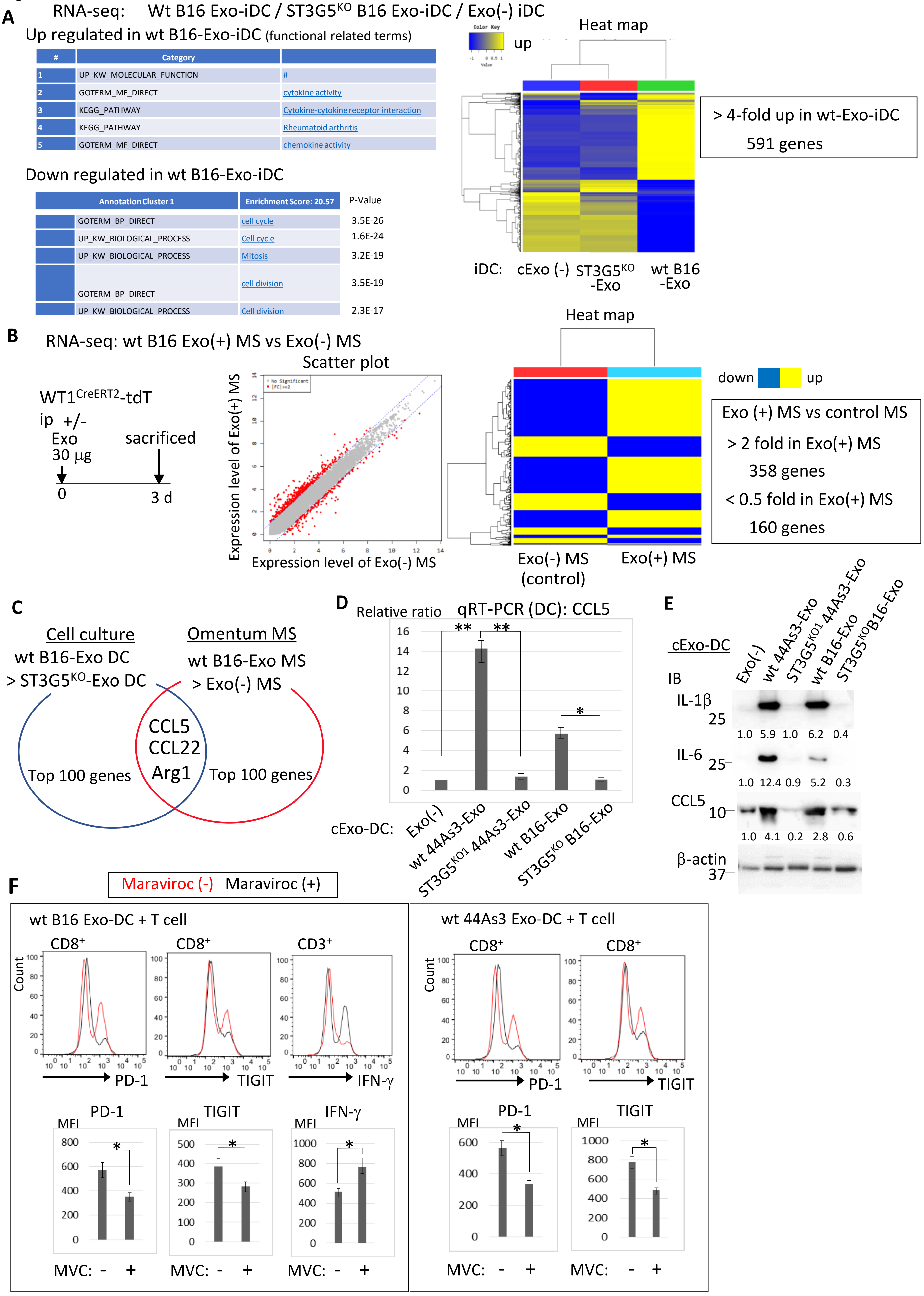
A
RNA-seq: Wt B16 Exo-iDC / ST3G5KO B16 Exo-iDC / Exo(-) iDC
Up regulated in wt B16-Exo-iDC (functional related terms)
| Category |
1 UP_KW_MOLECULAR_FUNCTION #
2 GOTERM_MF_DIRECT cytokine activity
3 KEGG_PATHWAY Cytokine-cytokine receptor interaction
4 KEGG_PATHWAY Rheumatoid arthritis
5 GOTERM_MF_DIRECT chemokine activity
Down regulated in wt B16-Exo-iDC
Annotation Cluster 1 | Enrichment Score: 20.57 | P-Value
GOTERM_BP_DIRECT cell cycle 3.5E-26
UP_KW_BIOLOGICAL_PROCESS Cell cycle 1.6E-24
UP_KW_BIOLOGICAL_PROCESS Mitosis 3.2E-19
GOTERM_BP_DIRECT cell division 3.5E-19
UP_KW_BIOLOGICAL_PROCESS Cell division 2.3E-17
Heat map
up
> 4-fold up in wt-Exo-iDC
591 genes
iDC: cExo (-) ST3G5KO -Exo wt B16 -Exo
B
RNA-seq: wt B16 Exo(+) MS vs Exo(-) MS
WT1CreERT2-tdT
ip +/- Exo 30 µg
sacrificed
0
3 d
Scatter plot
Expression level of Exo(+) MS
Expression level of Exo(-) MS
Heat map
down up
Exo (+) MS vs control MS
> 2 fold in Exo(+) MS
358 genes
< 0.5 fold in Exo(+) MS
160 genes
Exo(-) MS (control)
Exo(+) MS
C
Cell culture
wt B16-Exo DC
> ST3G5KO-Exo DC
Omentum MS
wt B16-Exo MS
> Exo(-) MS
CCL5
CCL22
Arg1
Top 100 genes
Top 100 genes
D
Relative ratio
qRT-PCR (DC): CCL5
cExo-DC: Exo(-) wt 44As3-Exo ST3G5KO1 44As3-Exo wt B16-Exo ST3G5KO B16-Exo
E
cExo-DC
IB
Exo(-) wt 44As3-Exo ST3G5KO1 44As3-Exo wt B16-Exo ST3G5KOB16-Exo
IL-1β 25
1.0 5.9 1.0 6.2 0.4
IL-6 25
1.0 12.4 0.9 5.2 0.3
CCL5 10
1.0 4.1 0.2 2.8 0.6
β-actin 37
F
Maraviroc (-) Maraviroc (+)
wt B16 Exo-DC + T cell
CD8+ CD8+ CD3+
Count
PD-1 TIGIT IFN-γ
MFI
MVC: - +
wt 44As3 Exo-DC + T cell
CD8+ CD8+
PD-1 TIGIT

Fig S7

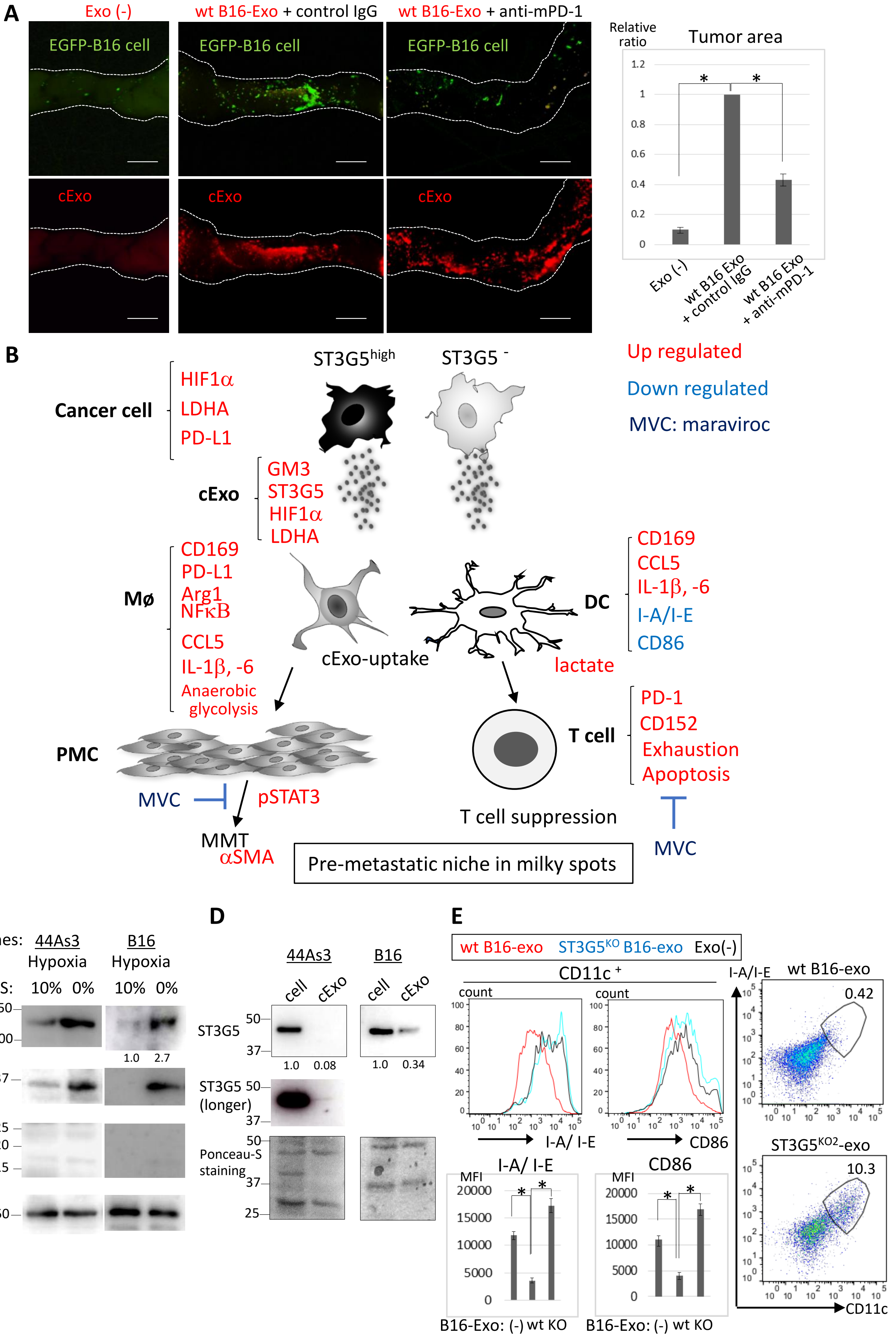
A
Exo (-)
wt B16-Exo + control IgG
wt B16-Exo + anti-mPD-1
EGFP-B16 cell
cExo
Relative ratio
Tumor area
1.2
1
0.8
0.6
0.4
0.2
0
*
Exo (-)
wt B16 Exo + control IgG
wt B16 Exo + anti-mPD-1
B
ST3G5high
ST3G5 -
Up regulated
Down regulated
MVC: maraviroc
Cancer cell
HIF1α
LDHA
PD-L1
cExo
GM3
ST3G5
HIF1α
LDHA
Mø
CD169
PD-L1
Arg1
NFκB
CCL5
IL-1β, -6
Anaerobic glycolysis
cExo-uptake
DC
CD169
CCL5
IL-1β, -6
I-A/I-E
CD86
lactate
PMC
MVC
pSTAT3
MMT
αSMA
T cell
PD-1
CD152
Exhaustion
Apoptosis
T cell suppression
MVC
Pre-metastatic niche in milky spots
C
Exosomes:
44As3 Hypoxia
B16 Hypoxia
FBS: 10% 0% 10% 0%
HIF1α
150
100
1.0
2.7
LDHA
37
Cleaved caspase3
25
20
15
CD63
50
D
44As3
B16
cell
cExo
ST3G5
50
37
1.0
0.08
0.34
ST3G5 (longer)
Ponceau-S staining
25
E
wt B16-exo
ST3G5KO B16-exo
Exo(-)
CD11c +
count
I-A/ I-E
CD86
MFI
20000
15000
10000
5000
B16-Exo: (-) wt KO
I-A/I-E
wt B16-exo
0.42
ST3G5KO2-exo
10.3
CD11c

Fig S8

A Immunochemical staining of ST3G5 in human gastric cancers

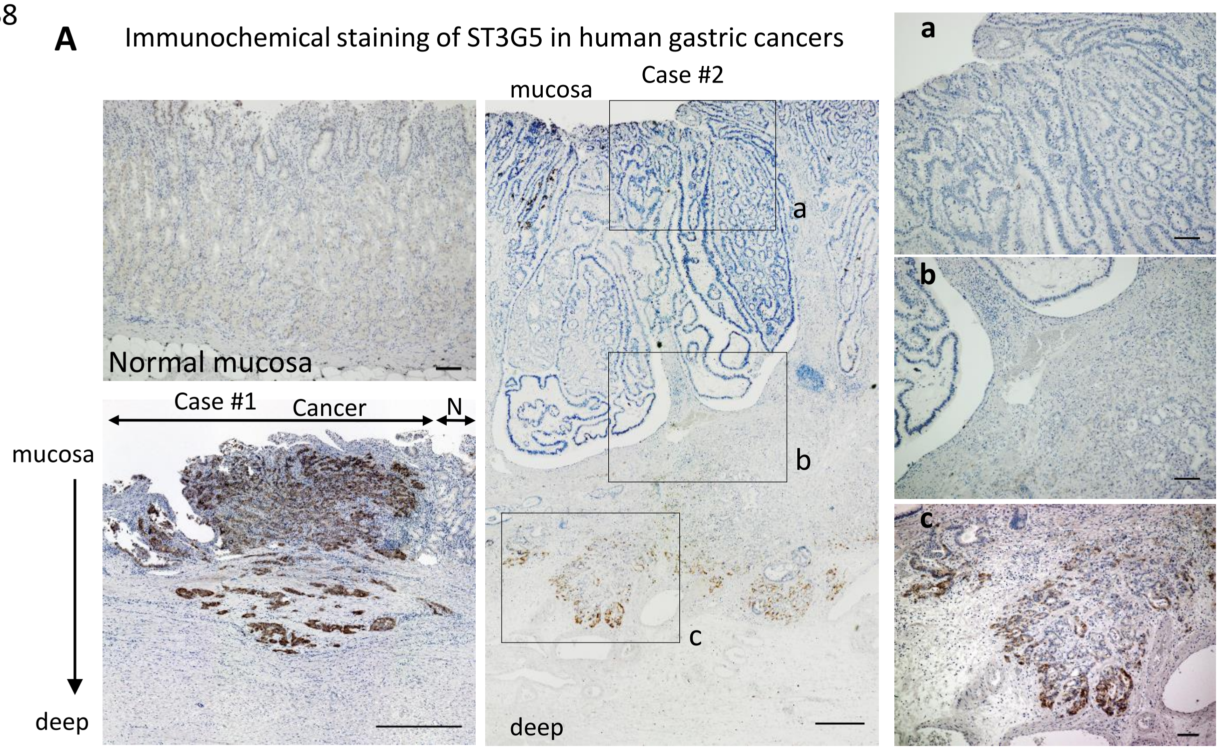

Incidence of ST3G5^{high} tumor

Total
29 % (7 / 24 cases)

Peritoneal relapse (+)
46 % (6 / 13 cases)

Peritoneal relapse (-)
9 % (1 / 11 cases)

Case #3 (peritoneal dissemination)

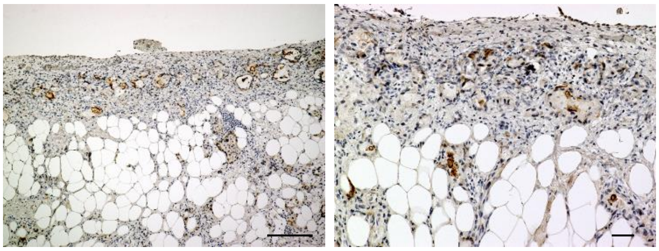

B Heat map

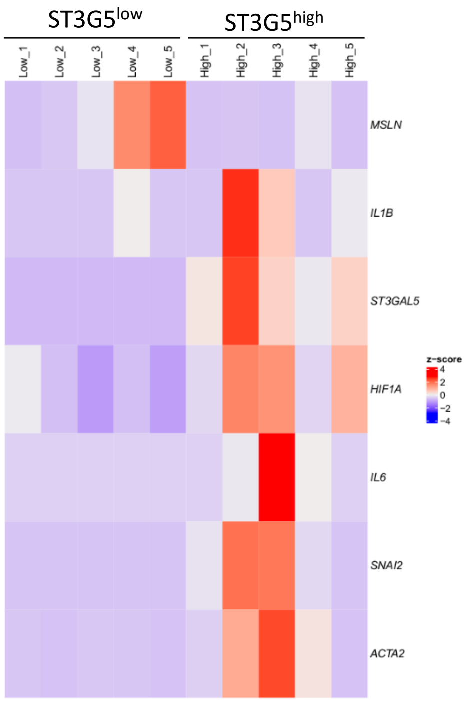

C

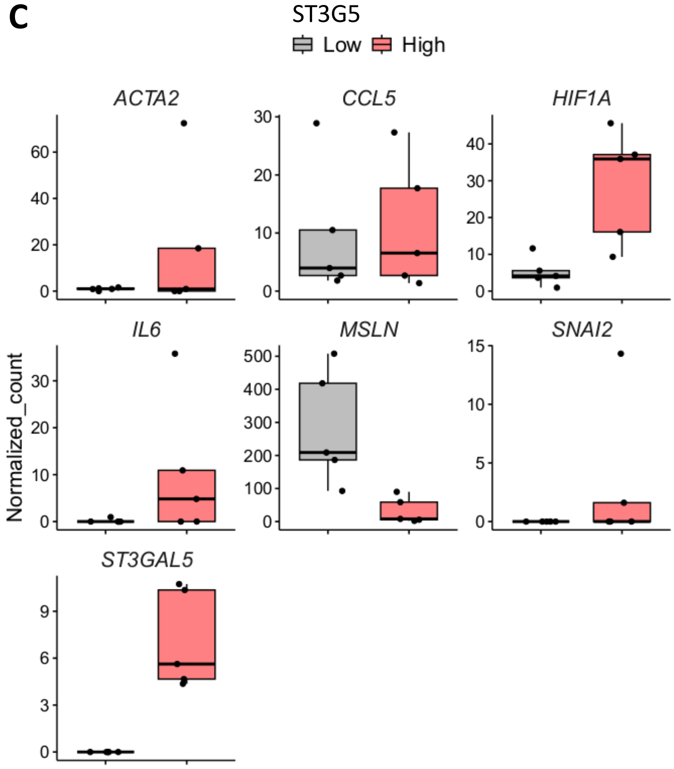

Supplement: Supplementary file 1 — Fig. S1. Expression of ST3G5 in cancer cell lines. Fig. S2. Effects of cExo on MΦs and PMCs. Fig. S3. Direct effects of ST3G5high‐cExo on T cells. Fig. S4. ST3G5high‐cExo attenuates T‐cell‐mediated cancer cell cytotoxicity. Fig. S5. ST3G5high 44As3‐cExo increased immune checkpoint molecules and T‐cell exhaustion. Fig. S6. RNA‐Seq analysis of cExo‐treated iDC and MS. Fig. S7. Model of ST3G5high‐cExo‐mediated premetastatic niche. Fig. S8. ST3G5 expression in human gastric cancer specimens. [file MOL2-18-21-s001.pdf]
